# Maternal attitudes and behaviours differentially shape infant early life experience: A cross cultural study

**DOI:** 10.1371/journal.pone.0278378

**Published:** 2022-12-21

**Authors:** Eve Holden, Joanna C. Buryn-Weitzel, Santa Atim, Hellen Biroch, Ed Donnellan, Kirsty E. Graham, Maggie Hoffman, Michael Jurua, Charlotte V. Knapper, Nicole J. Lahiff, Sophie Marshall, Josephine Paricia, Florence Tusiime, Claudia Wilke, Asifa Majid, Katie E. Slocombe

**Affiliations:** 1 Department of Psychology, University of York, York, United Kingdom; 2 School of Psychology and Neuroscience, University of St Andrews, St Andrews, United Kingdom; 3 Budongo Conservation Field Station, Nyabyeya, Uganda; 4 Department of Psychology, University College London, London, United Kingdom; 5 School of Human Evolution and Social Change and Institute of Human Origins, Arizona State University, Tempe, Arizona, United States of America; Obafemi Awolowo University, NIGERIA

## Abstract

Early life environments afford infants a variety of learning opportunities, and caregivers play a fundamental role in shaping infant early life experience. Variation in maternal attitudes and parenting practices is likely to be greater between than within cultures. However, there is limited cross-cultural work characterising how early life environment differs across populations. We examined the early life environment of infants from two cultural contexts where attitudes towards parenting and infant development were expected to differ: in a group of 53 mother-infant dyads in the UK and 44 mother-infant dyads in Uganda. Participants were studied longitudinally from when infants were 3– to 15–months-old. Questionnaire data revealed the Ugandan mothers had more relational attitudes towards parenting than the mothers from the UK, who had more autonomous parenting attitudes. Using questionnaires and observational methods, we examined whether infant development and experience aligned with maternal attitudes. We found the Ugandan infants experienced a more relational upbringing than the UK infants, with Ugandan infants receiving more distributed caregiving, more body contact with their mothers, and more proximity to mothers at night. Ugandan infants also showed earlier physical development compared to UK infants. Contrary to our expectations, however, Ugandan infants were not in closer proximity to their mothers during the day, did not have more people in proximity or more partners for social interaction compared to UK infants. In addition, when we examined attitudes towards specific behaviours, mothers’ attitudes rarely predicted infant experience in related contexts. Taken together our findings highlight the importance of measuring behaviour, rather than extrapolating expected behaviour based on attitudes alone. We found infants’ early life environment varies cross-culturally in many important ways and future research should investigate the consequences of these differences for later development.

## Introduction

Early life experiences can affect infant learning opportunities and behavioural development—and parents and caregivers play a large role in shaping these early life experiences. There is considerable cultural variation in parenting practices, for example, parents in different cultures vary in their feeding practices and how they promote motor development and socialisation of infants [1–6]. There is broad agreement that being brought up in different societies affects infant development (e.g. [1, 3, 4, 6–11]), but there is limited quantitative work characterising the naturalistic context in which infants develop and how this may be similar or different across populations [12–14].

Cross-cultural differences in parental behaviour, which can drive differences in infant experience, may be underpinned by varying attitudes towards parenting and infant development. One broad distinction made in the literature is between parenting attitudes which value interdependence or those which value independence. These attitudes differentially align with a *relational* (*‘Proximal’*) model or an *autonomous (‘Distal’)* model of parenting respectively [4, 9, 15–18]. Rural agricultural communities are said to adopt a more relational model where social context (e.g., social hierarchy, interpersonal relationships) and body contact are considered important [4, 9, 15–18]. Infant socialisation within this model stresses obedience and infants’ relations to others, and infants who have parents with relational attitudes develop compliance earlier than those who experience less relational parenting [9, 10]. Body stimulation of the infant to promote early motor development is also considered an important parenting practice in many rural agrarian societies [4]. The autonomous model, on the other hand, stresses object-based stimulation, face-to-face contexts and mutual gaze, agency, independence, and competition, and is common in Western, Educated, Industrialised, Rich, and Democratic (or ‘WEIRD’ [19]) societies [4, 9, 15–18]. Parents with autonomous attitudes stress infant self-development and autonomy, and their infants develop self-recognition earlier than those who experience less autonomous parenting [9, 10]. Although parental attitudes have been found to differ across cultural contexts, some researchers have argued that using a simplistic relational-autonomous dichotomy is theoretically inadequate and does not capture the empirical data appropriately [20, 21]. For example, these value systems may be better understood as probabilistic rather than deterministic. People within a community can ascribe to aspects of both autonomous and relational models, and the relative endorsement of each can change at different developmental time-points, all of which has led to calls for further studies relating both micro- and macro-factors [21]. In this paper, we examine the correspondence between attitudes and parental behaviour and the subsequent influence of each on infants’ early life experience.

Body stimulation of infants by caregivers is considered an important parenting practice in interdependent cultural models [4] and can encourage earlier physical development [1, 3, 22, 23]. Physical development affords infants new learning opportunities [24–26] with the attainment of physical milestones having downstream effects on key areas of socio-cognitive development. As infants develop motor skills that, for example, enable sitting and walking, their viewpoint of the world changes [27–31]. When infants are very young, their experiences are largely determined by their carers. Once infants develop a stable sitting position, they are able to conduct bimanual exploration of objects which builds their understanding of object properties [32–34]. Stable sitting also facilitates social looking and interaction [35, 36]. Once infants learn to crawl and walk, they gain new opportunities for who they can socialise with and how they can interact with their environment [31]. Walking enables infants to more easily select objects and carry them to social partners [37], which in turn facilitates triadic interactions between infant, partner and object—a key social skill linked to other complex social abilities [38–42]. Infant physical development seems to influence social development, and parenting practices can influence the age at which infants reach physical milestones [7, 22, 23].

The social environment clearly plays an important role in early life experience, and parental behaviour and attitudes can influence the social environment both directly and indirectly. Relational parenting models emphasise the importance of social relationships and are often associated with distributed childcare, which may have direct effects on the infant’s social environment. In addition, as outlined above, earlier physical development increases the opportunities for varied social interactions, highlighting an indirect route through which parental behaviour may influence the infant’s social environment. Social interactions have clear benefits for infant learning [41, 43], for example, in contrast to solo object play, social play with objects can promote more advanced behaviours through scaffolding [44]. Social learning is another important way for infants to gain information about the world around them: infants show social orientation from birth [45], and can learn from observation of others interacting with each other and the environment (e.g., how to use objects, local social norms, and words [46–49]). However, opportunities for social learning may be constrained by the number of people who tend to be in the vicinity of the infant.

The literature shows that infant early life experience can affect learning and social development, but it falls victim to the persistent sampling bias in developmental psychology of being based on limited, mostly WEIRD populations ([50]: *North America* [28, 30, 31, 33, 34, 36, 44]; *Western Europe* [22, 27, 32, 51]). This is particularly problematic because it fails to capture the diversity of parenting models across societies. Moreover, the cross-cultural research that is available shows important developmental differences across diverse populations that are likely mediated by parenting practices. For example, the age of reaching physical milestones such as sitting or walking varies across children from different cultural settings [3, 6, 52, 53]. This variation has been linked to parenting practices such as diaper use and infant handling practices, such as physical stimulation, stretching, and postural support [1, 3, 6, 7]. In addition, Bornstein *et al*. [54] found that parents from 11 different societies, including WEIRD and non-WEIRD cultures, encouraged exploration of the physical environment at different rates, which correlated with cross-cultural differences in exploration of the environment by infants. They also found cross-cultural differences in infants’ social behaviour (e.g., looks to their mother, smiling, communication) and mothers’ social behaviour (e.g., nurturance behaviour, communication, expression of affection, and social play). Thus, infants from one culture may experience different early life environments to those from another culture as a result of developing physical skills at different rates, and/or engaging with caregivers who hold different parenting attitudes, and consequently interact and scaffold behaviours differently.

Previous cross-cultural research has provided some important insight into how the early life environment of infants varies in different societies, however, there are several important areas which remain unexplored. Firstly, the contribution of non-mothers (e.g., other adults, children) to infant early life experience is poorly understood. Landau [55] found that in some cultures the proportion of overall social stimulation received from the mother decreases as infants age, and by the end of their first year over half of infants’ social stimulation during play is with non-mother individuals. Other researchers also found the composition of children’s social opportunities changes with age [56, 57]. These findings highlight the important role non-mothers may play in an infant’s social environment, and how their contribution to the infant’s environment might change over time. Infants in different cultures can also have different opportunities for socialisation with non-mothers [58], which further emphasises the importance of considering infants’ socialisation with non-mothers in cross-cultural research. However, to date in most of cross-cultural research of socialisation, there is a strong focus on mother-infant interactions [9, 10, 16, 18, 54], and most studies are limited to a single age point [9, 10, 16–18, 54]. Research which considers infants’ social interactions with both mother and non-mother individuals over the first years of life would add to our understanding of infant early life social environment.

Finally, while some may expect cultural attitudes to match up to behaviours, human attitudes, intentions, and behaviours do not always align (e.g., sustainability, health behaviours [59–63]) and prior research has shown parental attitudes or self-reports do not always match up with actual parenting behaviours (e.g., [64–67]). Most studies examining autonomous and relational parenting focus either on attitude questionnaires or observations of behaviour in a single context (often mother-infant play) [9, 10, 16–18]. It is therefore unclear whether parental behaviour in play contexts is representative of their behaviour in other contexts, and few previous studies have linked parental autonomous-relational attitudes to expressed behaviour.

In summary, differences in infants’ physical development and social environment afford different learning opportunities, and thus the early life environment is important for understanding the context in which infants develop. However, previous research is limited in its cross-cultural applicability: thus far, cross-cultural research concerning caregiver influences on the early life environment tends to focus exclusively on mother-infant relations, and is often limited to single age-points. Furthermore, these studies often either focus on a single behavioural context, or only report parental attitudes without linking them to behaviour. The main aim of this study was to bring together different aspects of early life environment which could be linked to infant learning and socialisation, using a longitudinal, mixed methods approach (i.e. using self-report questionnaires and observational methods), to give a rich cross-cultural description of the early life environment and maternal attitudes in two different cultural groups.

### Study outline and hypotheses

Using observational methods and questionnaire data, we present a case study comparison following infants between 3– and 15-months of age in two cultural contexts in the UK and Uganda. The UK participants were mother-infant dyads with limited ethnolinguistic diversity, living in or close to the city of York in the UK, who relied on income to sustain themselves (similar characteristics to groups typically categorised as having autonomous parenting: cf., [4, 68]). The Ugandan participants were mother-infant dyads with large ethnolinguistic diversity, living in or close to villages within the Nyabyeya parish, a rural area of Uganda, where subsistence farming is common (with characteristics more similar to groups typically categorised as having relational parenting: cf., [4, 68]).

In order to address our aims, we first determined whether we were justified in considering participants from the UK and Uganda as two distinct groups in terms of their maternal attitudes and infant early life experience. As parenting practices can vary across small geographical regions [69, 70], we checked for natural clusters of data that aligned with the ethnolinguistic groups within the Ugandan sample, to avoid making an assumption that the geographically proximal participants were a single homogenous culture for the purposes of our investigation. Second, we examined questionnaire data to characterise the maternal attitudes towards parenting and their socialisation goals for their infants. It is important to empirically verify attitudes, given previous critiques of dichotomous categorisation of populations (e.g., [20, 21, 66]). We predicted that mothers from the UK sample would have more autonomous attitudes, and those from the Ugandan sample would have more relational attitudes given the similarities of these groups to previously described groups (cf., [4, 68]).

We then examined measures of infant early life experience and maternal behaviour, and predicted that several aspects of this would differ with infant age, cultural group, and mothers’ expected alignment to autonomous or relational parenting models. More specifically, given previous research with agrarian societies and interdependent cultural models, it would be predicted that mothers from rural Uganda emphasise physical stimulation of their infants and (i) their infants reach physical milestones at earlier ages than infants of parents from the UK [4, 6, 22, 23, 54, 71]. It would also be predicted that (ii) the group of Ugandan infants would have a larger number of caregivers than the UK infants, as distributed child care is associated with more relational parenting models [15]. Since parents with relational attitudes also value the social context (e.g., social hierarchy, interpersonal relationships, and group goals) more than those with autonomous attitudes [4, 9, 15, 17, 18, 72], and household sizes were larger in Uganda, we predicted, in terms of social environment and interactions that (iii) Ugandan infants would have more people in proximity and (iv) although rates of social activity including play would be similar in UK and Uganda, Ugandan infants would have more social partners than UK infants. We also expected that (v) as infants aged they would experience more variety in their social interaction partners [22, 55]. Since parents with more autonomous attitudes promote independence in infants, and those with more relational attitudes value more prompt responses to infant cues, we expected (vi) less proximity between mother and infant both during the day and night in the UK sample in comparison to the Ugandan sample. As relational parenting values stress body contact, we expected (vii) more mother-infant body contact in the Ugandan sample, especially during play, but with a reduction in body contact in both groups as infants age and become more independent.

Finally, we examined the correspondence between specific attitudes and behaviour in UK and Ugandan mothers at an individual level, to test whether behaviour would match attitudes.

## Methods

### Participants

Participants were 53 mother-infant dyads from the UK (25 female and 28 male infants; 4 of who were two pairs of twins), and 44 mother-infant dyads from Uganda (24 female and 20 male infants; 1 twin). We extend our condolences to the family who lost one of their twins at 12-months-old, and we do not include data for the infant who passed away. Two Ugandan mother-infant dyads were excluded from this study because limited data was collected before they discontinued participation in the study.

Participants were part of a larger longitudinal study of early social cognition (3–24 months), however the present study focuses on infants up to 15–months-old (Covid-19 disrupted data collection from 18 months onwards). Participants for the UK sample were recruited in the York area through adverts at local children centres, adverts on York Mumbler (a website advertising events and opportunities for parents in York), a Facebook page advert, researcher presentations at baby sensory classes explaining our project, and through word of mouth. Participants were recruited during the mother’s pregnancy or when the infant was between 0– and 5–months-old. Participants for the Ugandan sample were recruited in the Nyabyeya parish, Masindi district, Uganda. Mothers, pregnant women, and interested individuals were invited to local information meetings. Invitations were made verbally at village and church meetings and further disseminated through word of mouth. Women who were interested in participating in the study were asked to register the birth date of their infant with local researchers. Participants from this list were invited to join the study if the mother spoke one of three languages to her child for which we had a translator available (Alur, Lugbara, or Swahili), and if the family lived close to the research station (within ~10 km). In periods where we had multiple eligible infants born close together, we prioritised maintaining a balance of male and female infants and first born and non-first born infants in the sample. Participant demographic information is summarised in Table 1.

**Table 1:** Sample demographics.

Demographic variables		UK	Uganda
Ethnolinguistic information	Mother nationality	49 mothers were born in UK, 1 in Romania, and 1 in Australia. All 51 were raised in the UK	For 39 mothers where information was available, 34 were born in Uganda, 4 in Democratic Republic of Congo, and 1 in Sudan. 41 mothers were raised in Uganda. 2 were raised in Congo. 1 relocated from Congo aged 8.
	Mother ethnicity	42 white British, 1 mixed British, 7 British (no further specification), 1 undisclosed	Of 41 mothers where information available: 16 Alur, 13 Lugbara, 3 Banyoro, 3 Kakwa, 2 Balendru,1 Akebu, 1 Kaliko, 1 Madi, 1 Acholi/Alur.
	Languages spoken to infant by parents	All mothers only spoke English with the study infants. Two fathers spoke an additional language with their infant (one French and one Irish).	Mothers spoke a median of 2 languages (range = 1–5) with infants. Of 44 mothers, 39 spoke Swahili, 24 spoke Alur, 9 spoke Lugbara, 6 spoke Runyuro, 6 spoke English, and 1 spoke Kakwa. Of 41 fathers where data were available, 12 spoke an additional language with their infant, and 2 spoke an additional two languages with their infant.
Socioeconomic status (SES)	Mean parental Hollingshead SES score [73] (possible range = 0–66)	*M* = 53.5 (*SD =* 10.3; range = 24.5–66.0; available *n* = 50).	*M* = 8.2 (*SD =* 6.4; range = 0–28; available *n* = 38).
	Mother literacy	All mothers could read and write in English.	Of 38 mothers where information was available, 11 could read and write in at least one language; 13 reported some level of reading or writing skills; 14 could not read or write.
	Mother’s highest level of education	Seven mothers’ highest education was secondary-school qualifications (or equivalent, e.g., A-levels, GSCEs), 23 had undergraduate degrees (or equivalent), and 21 had postgraduate qualifications.	Eight mothers had no education, 29 had some primary school education, and 7 had some secondary school education.
	Family income source	12% of mothers had no profession. All fathers had a profession.	Many Ugandan families were subsistence farmers with no external source of income. 91% of mothers, and 41% of fathers had no additional occupation.

## Materials and procedure

### Structure of data collection

Full-Day Follows (described below) were conducted at five time-points: when the infants were approximately 3-months-old (if they had been recruited before this time), 6–, 9–, 12–, and 15–months-old. Four questionnaires were used in this study: a Background Questionnaire, a Developmental Questionnaire, a Parenting Practices Questionnaire, and a Socialisation Goals Questionnaire. The Background Questionnaire and Developmental Questionnaire were presented at the same five time-points as the Full-Day Follows. Parenting Practices and Socialisation Goals Questionnaires were administered at 11 months. See Table 2 for sample size, and summary of infants’ age when data was collected.

**Table 2:** Mean infant age, spread, and sample size for participants included at each time-point.

	Full-Day Follows						Questionnaires					
	UK			Uganda			UK			Uganda		
Time-point	Age in months *M (SD)*	Range	*n*	Age in months *M (SD)*	Range	*n*	Age in months *M (SD)*	Range	*n*	Age in months *M (SD)*	Range	*n*
3 months	3.1 *(0*.*34)*	*2*.*5*–*4*.*0*	39	3.3 *(0*.*30)*	*2*.*6*–*3*.*8*	31	2.9 *(0*.*34)*	*2*.*5*–*3*.*8*	39	3.1 *(0*.*34)*	*2*.*2*–*3*.*7*	38
6 months	6.0 *(0*.*25)*	*5*.*2*–*6*.*4*	53	6.2 *(0*.*20)*	*5*.*8*–*6*.*5*	35	5.9 *(0*.*29)*	*5*.*3*–*6*.*6*	53	6.0 *(0*.*27)*	*5*.*6*–*6*.*5*	42
9 months	8.9 *(0*.*26)*	*8*.*5*–*9*.*6*	50	9.3 *(0*.*23)*	*8*.*8*–*9*.*9*	40	9.0 *(0*.*27)*	*8*.*5*–*9*.*5*	51	9.1 *(0*.*28)*	*8*.*6*–*9*.*9*	43
11 months	-	-	-	-	-	-	11.0 *(0*.*26)*	*10*.*5*–*11*.*7*	51	11.3 *(0*.*28)*	*10*.*6*–*12*.*3*	41
12 months	12.0 *(0*.*53)*	*11*.*5*–*14*.*9*	48	12.4 *(0*.*19)*	*12*.*0*–*13*.*0*	38	12.0 *(0*.*31)*	*11*.*1*–*12*.*9*	49	12.2 *(0*.*29)*	*11*.*6*–*12*.*9*	40
15 months	15.0 *(0*.*27)*	*14*.*4*–*15*.*6*	45	15.1 *(0*.*26)*	*14*.*6*–*15*.*6*	42	14.9 *(0*.*42)*	*14*.*2*–*16*.*4*	49	15.1 *(0*.*19)*	*14*.*6*–*15*.*5*	42

Note 1: Participants were excluded from analyses if their data were collected outside one month either side of their time-point month birthday.

Note 2: The Background Questionnaire and Developmental Questionnaire were collected at 3, 6, 9, 12 and 15 months (not collected at 11months). Data for the Parenting Practices Questionnaire and Socialisation Goals Questionnaire were collected only at 11 months.

### Translation of materials in Uganda

Local researchers were trained as a group in the delivery of the information sheet, consent form, the Background Questionnaire, and Developmental Questionnaire. The researchers reached consensus on the most appropriate way to translate and explain the content in Alur, Lugbara, and Swahili.

For the Socialisation Goals and Parenting Practices Questionnaires, consistency in phrasing was deemed critical because slight differences may influence interpretation. These questionnaires were translated into Alur, Lugbara, and Swahili by local researchers fluent in English and the respective language. Translations were then back-translated into English by a different local researcher and the meaning checked by a native English speaker (PhD student or post-doctoral researcher). If the intended meaning was not fully retained, then the process was repeated until a satisfactory translation was secured. If after repeated attempts at translation an item was not satisfactorily conveying the intended original meaning, it was deemed unsuitable and dropped from the questionnaire. To ensure consistency in delivery of statements across participants, voice recordings of questions in Alur, Lugbara, and Swahili were made. The recordings were stored on a smartphone and presented to participants via a small portable Bluetooth speaker.

### Questionnaires

In order to familiarise participants with Likert scale questions we also administered a Warm-up Questionnaire (see S1 Methods in S1 File), but no data from this questionnaire were analysed.

The Background Questionnaire had 50-items covering background and demographic information for the participants, including topics such as parents’ ethnicity, education, languages spoken, household members, infant caregivers, and infant sleeping and feeding habits (adapted from the work of Kaller [68]). We extracted information about the mother’s ethnolinguistic group and infant sleeping arrangements for analysis.

A 26-item Developmental Questionnaire was used to record the developmental milestones infants had reached, such as recognising family members, as well as physical and communicative milestones. Mothers were asked to indicate which abilities their infant showed at each age point. The abilities of interest to this study were: sitting without support, crawling, and walking alone a few steps.

A Parenting Practices Questionnaire was composed of 10 statements from the work of Keller and colleagues ([4, 17]) plus 29 other questions designed by Kaller and Slocombe (unpublished data) on what mothers consider appropriate parenting behaviour. Each statement was accompanied by a 5-point Likert scale ranging from strongly disagree to strongly agree. The ten Keller [4] statements (Table 3) and one Kaller and Slocombe statement “*It is important to devote a lot of time exclusively to the baby*” (only presented to mothers in the UK as this question could not be successfully translated i.e. meaning not retained in back translation) were analysed in this study.

**Table 3:** Ten Parenting Practices statements [4, 17] and their categorisation according to Keller [4] as autonomous or relational.

Parenting Practices	Autonomous or Relational
1. It is important to rock a crying baby in your arms in order to console him/her.	Relational
2. Sleeping through the night should be trained as early as possible.	Autonomous
3. It is not necessary to react immediately to a crying baby.	Autonomous
4. It is never too early to direct the baby’s attention towards objects and toys.	Autonomous
5. Babies should be encouraged to be as physically active as possible so that they become strong.	Relational
6. If a baby is fussy, he/she should be picked-up immediately.	Relational
7. It is good for a baby to sleep alone.	Autonomous
8. When a baby cries, he/she should be nursed immediately.	Relational
9. Babies should be left crying for a moment in order to see whether they console themselves.	Autonomous
10. A baby should always be in close proximity with his/her mother, so that she can react immediately to his/her signals.	Relational

Note: The instructions given to mothers when their infants were 11–months-old, were “Please think of a baby of about 11 months of age and express your agreement or disagreement with these statements”.

Mothers were also given a Socialisation Goals Questionnaire, composed of 10 statements (from [4]). Two autonomous statements could not be appropriately translated/back translated into the Ugandan languages (“*During the first three years of life it is really important that children develop a sense of self*”; “*During the first three years of life it is really important that children develop a sense of self-esteem*”). As these statements could not be presented in Uganda, they were excluded from analysis in this study. The statements analysed are shown in Table 4. We chose to assess maternal socialisation goals in two different ways: in the first part of the questionnaire, mothers’ agreement with each statement was measured. Mothers were presented with each of the eight statements at a time and asked to indicate their agreement on a 5-point Likert scale 1 = ‘strongly disagree’ to 5 = ‘strongly agree’). The general instructions for this part of the questionnaire were as follows: *“The following statements describe different characteristics that children should acquire during the first three years of their life*. *We would like you to indicate how much you agree with each of these statements”*.

**Table 4:** Table of analysed Socialisation Goals statements and their categorisation according to Keller [4] as autonomous or relational.

Socialisation Goals	Autonomous or relational
During the first three years of life it is really important that children…	
learn to cheer up others	Relational
learn to obey elderly people	Relational
learn to obey parents*	Relational
learn to care for the well-being of others*	Relational
learn to control emotions*	Relational
develop competitiveness*	Autonomous
develop self-confidence*	Autonomous
develop independence*	Autonomous

Note: These were administered in two ways: (i) mother agreement with each statement using a 5-point Likert scale and (ii) choice of the most important goal in pairwise comparisons between relational and autonomous statements (statements included in the pairwise comparisons are indicated with an asterisks (*)).

In the second part of the questionnaire, we aimed to measure the mothers’ choices of the most important goals and therefore presented mothers with a forced choice task. They were given pairs of statements consisting of one relational and one autonomous goal at a time. Mothers were asked to choose the more important one (even in cases where they agreed or disagreed with both statements). As we had only been able to translate three autonomous statements into the Ugandan languages, we selected three out of the five relational items for this part of the questionnaire in order to have an equal number of statements per category. Each of the three autonomous items was compared with each of the three selected relational items (Table 4) which resulted in a total of nine pairwise comparisons.

We extracted three measures of alignment with relational/autonomous goals. First, each of the ten Keller [4] statements from the Parenting Practices Questionnaire and all statements from the Socialisation Goals Questionnaire were categorised as relating to relational or autonomous parenting attitudes (Tables 3 and 4). We then extracted a measure for each of these questionnaires separately. We calculated the mean Likert rating each participant gave to the sets of autonomous and relational questions respectively to create a mean autonomous score and a mean relational score for each participant. UK mothers were more likely to use the full spectrum of the Likert scale compared to Ugandan mothers, who more often used extremes of the scale (i.e., strongly disagree or strongly agree). To mitigate the effects of response style, we calculated a ‘difference score’ to characterise the relative relational and autonomous parenting attitudes for each participant (instead of characterising mothers relational and autonomous views separately [4, 74, 75]). The difference score was a participant’s mean autonomous score minus their mean relational score (with potential values ranging from -4 to +4; values > 0 categorised opinions as overall ‘more autonomous’ and values < 0 as overall ‘more relational’).

Lastly, we used the forced-choice task with the Socialisation Goals statements (Table 4) to calculate whether mothers valued relational or autonomous statements as more important. Each statement chosen by the mother was given a score of +1, and those not chosen were given a score of -1. For each mother, we calculated the mean score for relational items in each of the nine pairwise comparisons. A positive mean score indicated the mother aligned more with relational than autonomous socialisation goals. A negative mean score indicated stronger alignment with autonomous socialisation goals. A score of zero indicated the mother did not consistently chose relational or autonomous statements as more important. An item-level analysis of the six statements included in the forced choice task is described and reported in S5 Methods and S2 Results in S1 File.

### Questionnaire data collection procedure

In the UK, questionnaire data were collected as part of a 1.5 to 2 hour research session at the participants’ homes, usually run by two researchers. UK mothers were asked to fill in hard copies of all questionnaires at a convenient point in the visit.

In Uganda, questionnaire data were collected as part of a 2 to 4 hour research session at the participants’ homes, with at least two researchers (including at least one local research assistant). The local researcher had at least one fluent language in common with the mother so they could also act as translator. Due to low literacy rate in our Ugandan mothers, the Background Questionnaire and Developmental Questionnaire were presented verbally to mothers. Mothers responded verbally, and answers were then recorded on hard copies of the questionnaires by a researcher. Socialisation Goals Questionnaires, Parenting Practices Questionnaires, and Warm-up Questionnaires were presented by playing the voice recording of the statements to the participants. The participants verbalised their response and the researchers recorded answers on hard copies of questionnaires.

The Warm-up Questionnaire was always presented before the Parenting Practices Questionnaire and Socialisation Goals Questionnaire so that mothers could become familiar with the format. There was no fixed order for the presentation of other questionnaires during the visit.

### Observational data collection procedure: Full-day follows

Observational data was collected from a sample day of the mother-infant dyads’ life. Throughout eight hours of this Full-Day Follow, we implemented instantaneous scan sampling [76], to collect information regarding the mothers’ and infants’ activities and social environment. Using this type of sampling, the social environment and the behaviour the mother and infant were engaging in at specific points in time were recorded, providing a series of snapshots of behaviour over the day. Mothers were encouraged to select a day where they or another family member was caring for the child (see S2 Methods in S1 File for further details on choice of Full-Day Follow). Mothers were asked to behave as normal throughout the day and not to change their plans or behaviours to accommodate data collection. For each scan sample we recorded the mothers’ and infants’ activity and categorised behaviours according to a standard list of behaviours (see S3 Methods in S1 File), with analyses focussing on social activities including play. For any social activities (e.g., play, being fed), the identity of the social partner was recorded and later categorised as mother, non-mother adult, or child. As a measure of infants’ immediate social environment, the number, and where possible age category (child or adult), of people within five metres of the infant was recorded too, as well as the infant’s proximity to their mother.

In Uganda, Full-Day Follow data were collected by local research assistants via direct observation, with scan samples collected every 15 minutes. The family was asked to ignore the research assistant, who observed the family in their compound as unobtrusively as possible. In the UK, none of our participants wished to be directly observed for a full day and so instead mothers were phoned and were asked to report the requested information every 30 minutes. If a UK infant was supervised by someone other than the mother, we asked these questions to this caregiver if they were available. Data sheets used for Full-Day Follow data collection are in S4 Methods in S1 File.

### Training and interobserver reliability for observational data

For UK participants, Full-Day Follows were conducted by a team of researchers. Training consisted of familiarisation with written materials that explained the data collection and categorisations of responses. Individuals then observed an experienced researcher conducting the Full-Day Follow phone calls and entering data into spreadsheets. New data collectors were then supervised by a more experienced researcher while they conducted their own first Full-Day Follow phone calls, and corrected if there were any departures from protocol. New data collectors were not permitted to collect data unsupervised until the trained collector was satisfied with their performance. To assess reliability of data collection across researchers, we recorded phone calls with four participants and asked researchers to listen to the calls and complete data sheets, including categorisation of responses. We identified the five researchers who collected the majority of the data and acted as trainers and their data was compared to that of JCBW (who lead UK Full-Day Follow data collection training), from the same recorded calls. The mean Cohen Kappa [77] scores were .74 or above for all variables used in this study, indicating data collection was reliable.

In Uganda, two local research assistants (PJ and AS) were trained in Full-Day Follow data collection by EH. Training consisted of familiarisation with written materials and then practice of data collection together in various imaginary situations, as well as real-life observations. After training, EH, PJ, and AS, conducted three days of visits with three different families. On each day they observed the same mother-infant dyad and recorded data without consultation with one another. The data collected by PJ and AS were compared to the data collected by EH for each variable, and Cohen’s Kappas [77] calculated. The mean Cohen’s Kappas were. 73 or above for both research assistants for all variables used in this study, indicating high inter-observer reliability.

Given the higher frequency of scan samples in Full-Day Follows in the Ugandan data set (15 minutes in Ugandan Full-Day Follows versus 30 minutes in UK Full-Day Follows), for any analysis using summary variables from a Full-Day Follow, the Ugandan data was split to create two sets of data (Set 1 and Set 2) with scan samples separated by 30 minutes. Set 1 or Set 2 of the Ugandan data was randomly chosen for each Full-Day Follow summary variable used in analyses.

### Ethics statement

Ethical approval was obtained for data collection from the University of York Psychology department Ethics Committee. Additionally, we obtained ethics permission from the Regional Ethics Committee at the Ugandan Virus Research Institute, and permits from the Ugandan National Council for Science and Technology for data collection conducted in Uganda. UK mothers read an information sheet, asked any questions they had and then provided written consent for their infant and themselves to participate. In Uganda, local research assistants verbally presented the information sheet and consent form in one of the three languages the mother was fluent in (Alur Lugbara, or Swahili as appropriate), the mothers asked any questions they had, then provided a thumbprint or written signature to indicate consent.

### Data analysis

Analyses were run in *R* version 4.0.1 [78] unless otherwise noted.

#### Do participants from UK and Uganda form two distinct groups?

A cluster analysis was conducted to determine a) if early life environment for infants fell into two distinct groups (dyads from the UK and Uganda), and (b) if early life environment for infants in Uganda fell into distinct groups based on ethnolinguistic group. To provide a holistic picture of infant early life environment, we included variables concerning mothers’ attitude towards infant independence and social environment, infant attainment of physical milestones and environmental exploration, and infant social environment and social interactions (see Table C in S6 Methods of S1 File for details of specific measures included). Prior to the cluster analysis, a principal component analysis (PCA) was conducted as a method of data reduction. We ran the cluster analysis on the dyad scores from the six extracted principal components (PCs), rather than the raw data, as this approach has several advantages: (i) shared variation across variables was the focus of clustering participants, and meant shared variation did not duplicate pull in variance accounted for by multiple variables, and (ii) no single variable would be responsible for any cluster formation if that variable was more consistent for certain groups of participants. This meant if one thing was (almost) completely distinct in the infant environment, this was not overly weighted towards forming a cluster; it was only if this variable had shared variance with other points of interest did it impact cluster formation. PCA analyses and score calculation details can be found in S6 Methods in S1 File.

A hierarchical cluster analysis was performed using IBM SPSS Statistics 28 [79] on the six PCs extracted. Between-groups linkage method was used, and squared Euclidean distance was used as the measure of similarity. To test whether participants formed two groups (UK and Uganda), we selected a two cluster solution and examined the resulting cluster membership of UK and Ugandan participants. To test whether Ugandan participants formed groups according to their ethnolinguistic group (Alur, Lugbara, other), we selected a 3 cluster solution and examined the cluster membership of Alur, Lugbara and other ethnolinguistic group participants. We then planned Chi Square tests to examine if the distribution of (i) UK and Ugandan participants and (ii) Alur, Lugbara and other ethnolinguistic group participants across clusters was significantly different to a random distribution. The other six ethnolinguistic groups in Uganda had 1–3 participants in, which was too few for inferential statistics, but we descriptively examined how they were related and distributed across clusters.

#### Do mothers from the UK and Uganda have different attitudes towards parenting and socialisation goals for their infants?

To test if UK and Ugandan mothers differed in their parenting and socialisation attitudes, three Kruskal-Wallis tests were conducted. The dependent variables in these three analyses were Parenting Practices Difference Score (score -4 to 4), Socialisation Goals Difference Score (score -4 to 4), and Relational Goal Choice Score (score -1 to +1) respectively. The independent variable in all tests was cultural group (UK or Uganda). Non-parametric tests were chosen due to non-normal distribution of the data and Kruskal Wallis tests were chosen over Mann-Whitney U tests due to unbalanced variance across groups.

#### Does cultural group and infant age influence early life environment?

Generalized Linear Mixed Models (GLMMs) were conducted using the lme4 package [80] to examine whether variation in infants’ attainment of physical milestones, their social environment, and social interactions (dependent variables), could be explained by cultural group, infant age, and the interaction between group and age (fixed factors). Infant age was entered in months (see S7 Methods in S1 File). The unit of analysis varied across models (see Table E in S8 Methods of S1 File) and included individual scan samples, whole Full-Day Follows, and questionnaire responses. As participants were sampled at multiple time-points and often contributed multiple data points for each time-point (e.g., individual scan samples), we entered participant identity as a random factor in all models. For models with a categorical (yes/no) dependent variable, a binomial error structure with a logit link function were implemented. For models with a dependent variable that generated count data (e.g., number of people in five metre proximity), either a poisson or negative binomial error structure was implemented. The full details of the factors, error structure, level of analysis (including sample size) and inclusion criteria for each model are provided in Table E in S8 Methods of S1 File.

Likelihood ratio tests were used to compare full models (with all fixed and random factors) and null models (models with only random factors) to determine whether the full model better explained variance in the data. To further understand interactions between infant age and cultural group, post-hoc models for each culture were conducted with the alpha level Bonferroni corrected for multiple comparisons. Overdispersion was checked for non-binomial GLMMs using an ‘overdisp test’ function provided by Roger Mundry. Model stability was checked by examining influential cases indicated by Cooks distance (calculated using the Influence.ME package [81]) greater than 4/*n* (where *n* = number participants). To understand the effect of influential cases, models were re-run excluding influential cases to see if it changed interpretation of the model (see S4 Results in S1 File). Where exclusion affected model interpretation, results of the alternate model are also presented.

#### Are parental attitudes associated with observed or reported parenting behaviour?

To test if maternal attitudes at 11 months were associated with observed or reported parenting behaviour at 12 months, we compared the behaviour of mothers who showed high and low agreement with the corresponding attitude question. Mothers who chose one of the two disagreement options or neutral were placed in the low agreement group and mothers who chose one of the two agreement options were placed in the high agreement group. For behavioural measures that produced binary categorical data (yes/no; e.g., does the infant share a bedroom?), Fisher’s exact tests were used to examine the distribution of high and low agreement mothers over the two behaviour categories. For behavioural measures that produced continuous data (e.g., proportion of scan samples where the mother is within five metres of infant), non-parametric Kruskal-Wallis tests were conducted to assess if the behaviour of the low and high agreement groups differed. As above, non-parametric Kruskall Wallis tests were chosen due to the non-normal distribution of the data and unbalanced variance across groups. Table F in S9 Methods of S1 File details the specific attitude questions and behavioural data used for these analyses.

## Results

### Participant characteristics

Characteristics of the infants’ home environment were extracted from questionnaires administered every three months–see Table 5. This table provides information about the context in which the study infants were being raised.

**Table 5:** Sample background information.

Variables		UK	Uganda
Mother background information	Mother age at birth of infant	*M* = 32.6 years (*SD* = 3.7; range = 25–41).	*M* = 27.0 years (*SD* = 7.0; range = 15–42).
	First time mothers	47% (including one set of twins).	23%
Household membership	Mean household size (including mother and infant)	*M* = 3.7 individuals (*SD* = 0.91; range = 3–8).	*M* = 6.4 individuals (*SD =* 2.8; range = 2–17).
	Household membership	Household members were consistent across the study for all except one mother-infant dyad.	For 82% of the participants, at least one person left or joined the household during the study period.
	Mother’s living arrangements	Mothers always lived with their infant.	Mothers always lived with their infant.
	Father’s living arrangements	For 98% of participants, the father always lived with the infant. For 2% participants the father lived with the infant full-time at some, but not all, of the time-points we visited the family.	For 55% of participants, the father always lived with their infant. For 25% of participants the father lived with the infant full-time at some, but not all, time-points we visited the family. For 20% of infants their father never lived in the infant’s home full-time (i.e. he stayed permanently elsewhere, or he had multiple homes).
	Mean number children (under 17-year-olds) living with the infant	0.7 children (*SD =* 0.75; range = 0–3).	2.8 children (*SD =* 1.95; range = 0–9).
	% infants who lived with at least one child under 5*	47.2%	*M =* 65.8%
	% infants who lived with at least one child aged 6–10 years*	9.4%	*M =* 67.0%
	% infants who lived with at least one child aged 11–16 years*	3.8%	*M =* 55.5%
Infant care	Paternal time with infant	Fathers were reported as caregivers for all infants. Averaged across all age-points, they spent a mean of 39.3 hours per week (*SD =* 18.3) with the infant.	Fathers were listed as a caregiver for at least one time-point for 41% of participants. Duration of time spent with infants was not available.
	Mother time separated from infant (e.g. while at work)	*M* = 12.9 hrs/week (*SD =* 10.6).	*M* = 7.8 hrs/week (*SD* = 8.9).
	Breast feeding	At 3 months: 81.1% At 6 months: 70.4% At 9 months: 56.4%, At 12 months: 50.0% At 15 months: 34.7%	At 3, 6 and 9 months: 100% At 12 months: 97.3% At 15 months: 90.2%
Home environment	Home structures	All UK mother-infant dyads lived in permanent structures with electricity. All had indoor bathrooms and mains plumbing.	Ugandan mother-infant dyads lived in mud or brick houses with straw or iron sheet roofs. Their homes consisted of a compound with two or more buildings for different purposes (e.g., sleeping, cooking). None had mains electricity but some had small personal solar panels. Water sources and latrines were outside the house.
	Indoor/outdoor living	Across time-points, mothers and infants on average spent more than half their time in daily activities indoors.	On average when infants were 3–6 months old they spent more than half their time in daily activities indoors, but from 9–15 months both mothers and infants spent less than half their daily activity time indoors.

Note: Participant background information was extracted from questionnaires administered every three months from 3–15 months.

Asterisks (*) indicate means were taken across time-points for Ugandan participants since household membership was dynamic. Means were not calculated in the UK sample since household children membership stayed stable during the course of data collection.

### Do participants from the UK and Uganda form two distinct groups?

The six Principal Components (PCs) extracted from the PCA explained 74% of variance in the data (see S1 Results in S1 File for PC loadings, eigenvalues, and variance explained by each component). Two cluster analyses were conducted on dyad scores calculated using the loadings of the original variables onto these six PCs (dyad score calculation details included in S6 Methods in S1 File). First, the cluster analysis of all participants revealed two clusters perfectly aligned with nationality of participants (i.e., 100% of UK participants belonged in cluster 1 and 100% of Ugandan participant belonged in cluster 2; S1 Results in S1 File). No Chi-Square test could be conducted on these classifications as they showed perfect separation into distinct clusters. The second cluster analysis was conducted over the Ugandan participants. Twenty-seven participants were assigned to cluster 1, fifteen to cluster 2, and two to cluster 3 (see S1 Results in S1 File). Given the small size of cluster 3, and low numbers of rarer ethnolinguistic groups, the alignment for these groups was not tested inferentially. Testing cluster membership for clusters 1 and 2 with Alur and Lugbara ethnolinguistic groups, the analysis revealed no significant association between cluster membership and ethnolinguistic group (see Figure E in S1 Results of S1 File; *χ*^2^_(1)_ = .697, *p* = .404). Descriptively, there was also no reliable clustering of members of rarer ethnolinguistic groups (1–3 participants) in our sample (see Table I in S1 Results of S1 File). As such, it was deemed appropriate for our research questions to regard the Ugandan data as one group in contrast to the UK group for subsequent analyses.

### Do mothers from the UK and Uganda have different attitudes towards parenting and socialisation goals for their infants?

**Fig 1:**
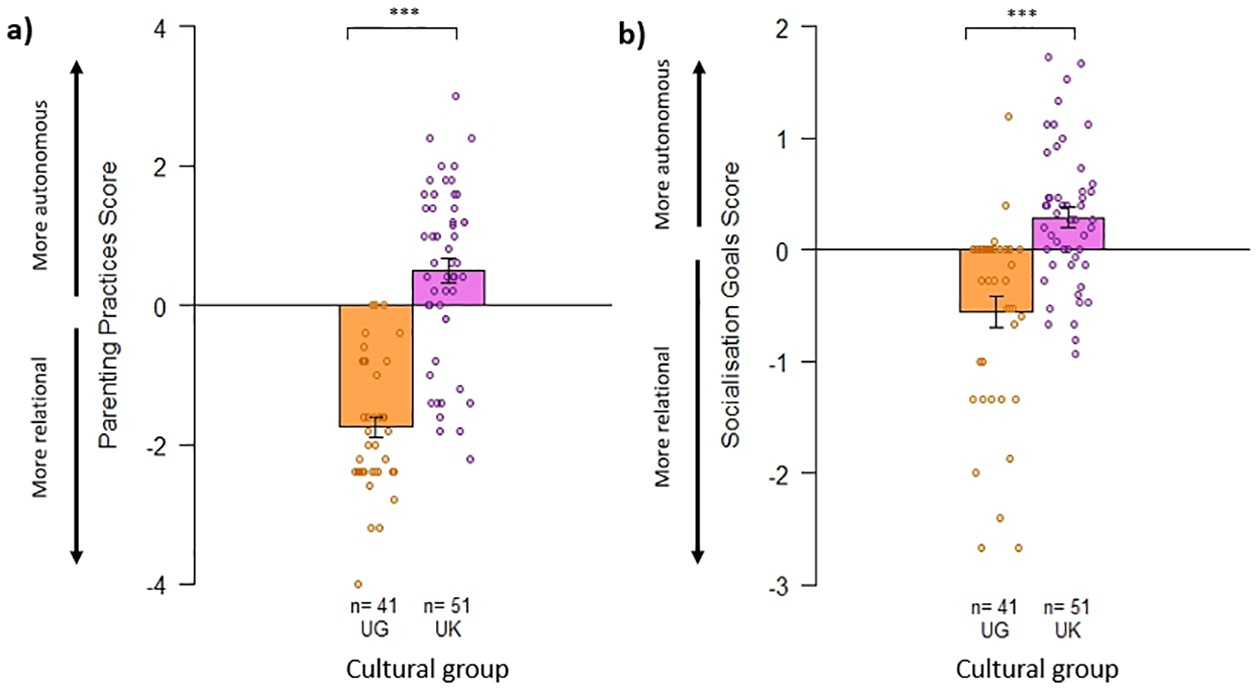
Graphs of difference scores for a) Parenting Practices, and b) Socialisation Goals attitudes. Note: Bars show mean difference score +/- SE bars. Points show individual participant difference scores and n shows the number of participants in each group. *** indicates significant difference at *p* < .001.

When considering difference scores in Likert scale agreement with relational and autonomous statements in Parenting Practices and Socialisation Goals Questionnaires, mothers’ attitudes were overall significantly more autonomous and less relational for UK than Ugandan participants (see Fig 1; Kruskal-Wallis tests: Parenting Practices: H_(1)_ = 47.4, *p* < .001; Socialisation Goals: H_(1)_ = 24.3, *p* < .001). However, the range within each group was larger than the mean difference between groups indicating there was substantial individual variation within communities.

In the forced choice comparisons, the median scores for relational goals were significantly lower in the UK mothers (Median = -0.11; IQR = -0.33 to 0.33) than Ugandan mothers (Median = 0.33; IQR = 0.11 to .78). This indicates UK mothers chose autonomous goals as more important than relational goals, to a greater degree than Ugandan mothers (H_(1)_ = 21.84, *p* < .001).

### Does cultural group and infant age influence early life environment?

GLMMs were conducted to establish if there was an effect of group or infant age on infant physical development, infant activities, infant carers, infant social environment, mother-infant relations, and infant social partners. For all GLMMs we report whether the full model explained more variance than the null model (Likelihood ratio test) and then report significant individual parameters. Full model results can be found in the S3 Results (Tables K-R) of S1 File.

#### Infant physical development

**Fig 2:**
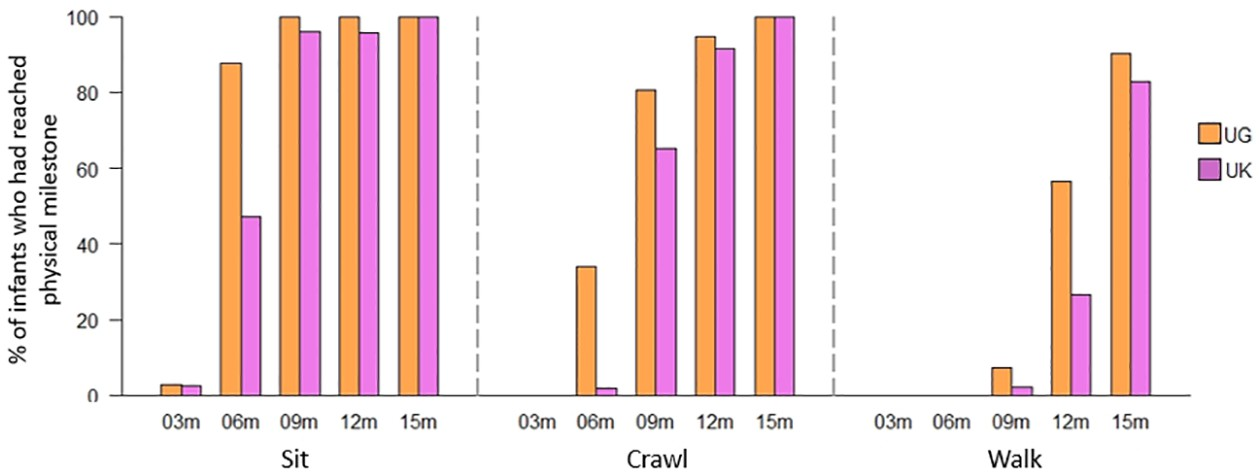
Percentage of infants who could (a) sit unsupported (b) crawl unsupported, and (c) walk unsupported, for each age and culture. Note: UG = Uganda. The number of Ugandan participants at time-points 3, 6, 9, 12, and 15 months was 37, 41, 41, 39, and 41 respectively. The number of UK participants at 3, 6, 9, 12, and 15 months was 39, 53, 49, 47, and 47 respectively, except for walking at 12 months where *n* = 45.

More UK infants reached physical milestones at later ages than Ugandan infants (Fig 2).

Overall, UK infants began being able to sit later than Ugandan infants (Likelihood ratio test: *χ*^2^_(3)_ = 6.95, *p* = .008; Group: Est = -1.30, *SE* = .458, *z* = -2.85, *p* = .004). UK infants were less likely than Ugandan infants to be able to sit from 2–10 months (with a probability of .39 that a child sampled in this age range from UK infants could sit, and a probability of .70 for Ugandan infants; Table K in S3 Results of S1 File).

Overall, UK infants began being able to crawl later than Ugandan infants (Likelihood ratio test: *χ*^2^_(3)_ = 12.7, *p* < .001; Group: Est = -1.39, *SE* = .419, *z* = -3.31, *p* <. 001). UK infants were less likely than Ugandan infants to be able to crawl from 5–13 months (with a probability of .53 that a child sampled in this age range from the UK Uganda could crawl and a probability of .82 for Ugandan infants; Table K in S3 Results of S1 File).

Overall, UK infants began being able to walk later than Ugandan infants (Likelihood ratio test: *χ*^2^_(3)_ = 6.03, *p* = .014; Group: Est = -1.07, *SE* = .504, *z* = -2.12, *p* <. 001). UK infants were less likely than Ugandan infants to be able to walk when from 8–16 months (with a probability of .23 that a child sampled in this age range from the UK could walk and a probability of .54 in Ugandan infants; Table K in S3 Results of S1 File).

#### Infant social environment—Caregivers

In both the UK and Uganda, mothers were the infants’ primary caregivers. Mothers were caregivers for 96% of scan samples in the UK and 79% in Uganda. UK infants had a lower number of carers in a day than Ugandan infants (Likelihood Ratio test: *χ*^2^_(2)_ = 106, *p* < .001; Group: Est = -1.59, *SE* = .347, *z* = -4.57, *p* < .001; Table L and Figure F in S3 Results of S1 File).). There was no effect of infant age on the number of carers an infant had and no significant interaction between group and age.

Non-mother adult caregivers were recorded at least once for 57% of UK participants and at least once for 91% of Ugandan participants. Fathers were recorded as a caregiver at least once for 37% of UK and 40% of Ugandan infants. In Full-Day Follows, 40% of non-mother caregivers in the UK were fathers and 24% of non-mother adult caregivers in Uganda were fathers. Infants were significantly less likely to have a non-mother adult caregiver during their Full-Day Follow in the UK than Uganda (Likelihood Ratio test: *χ*^2^_(2)_ = 12.6, *p* = .005; Group: Est = -1.05, *SE* = .428, *z* = -2.46, *p* = .014; Table L and Figure F in S3 Results of S1 File). There was no effect of age on how likely infants were to have a non-mother adult caregiver and no significant interaction between group and age.

No UK infant participants were ever recorded as having a carer who was below 17–years-old, whereas this was common in Uganda, seen at least once in all Ugandan participants and in 79% of Full-Day Follow days. In Uganda, children aged 5 years or younger were recorded as a caregiver at least once for 66% of participants, children aged 6–10 years were recorded as a caregiver at least once for 84% of participants, and children aged 11–16 years were recorded as a caregiver at least once for 64% of participants. There was a significant effect of infant age on the likelihood that a Ugandan infant was cared for by a child (Likelihood Ratio test: *χ*^2^_(2)_ = 7.03, *p* = .008; Age: Est = .270, *SE* = .100, *z* = -2.66, *p* = .008; Table L and Figure F in S3 Results of S1 File): as Ugandan infants aged, they were more likely to have a carer who was a child.

#### Infant social environment—People in proximity of infant

Next, we considered the availability of non-mother adults and children in proximity to infants. There was no effect of group or infant age on the number of non-mothers within five metres of an infant (Likelihood ratio test: (*χ*^2^_(2)_ = 3.99, *p =* .262).

As infants aged, the likelihood of having a non-mother adult within five metres decreased (Likelihood Ratio test: *χ*^2^_(2)_ = 22.2, *p <* .*001*; Age: Est = -.030, *SE* = .080, *z* = -4.11, *p* < .001; Table M and Figure G in S3 Results of S1 File). There was no effect of group on the likelihood of having a non-mother adult within five metres of the infant, and there was no significant interaction between group and age.

There was an interaction between group and infant age on how likely it was for a child to be within five metres of the infant (Likelihood Ratio test *χ*^2^_(2)_ = 20.8, *p <* .*001*; Age*Group: Est = .036, *SE* = .013, *z* = 2.88, *p* = .004; Table M and Figure G in S3 Results of S1 File). Although UK infants were less likely to have a child in proximity than Ugandan infants across all ages, the likelihood of being in proximity to another child varied more with age in the UK, whereas proximity to another child was relatively stable over time in Uganda (post-hoc models: UK age Est = .036, *SE* = .010, *z* = 3.43, *p* < .001; UG age Est = -.002, *SE* = .007, *z* = -.327, *p* = .743). However, nine UK participants and nine Ugandan participants were deemed overly influential in the GLMM indicating instability in the model. To further understand the effects of these participants, the GLMM was re-run without these participants. The model parameters were similar for the main effect of culture (model including all participants: group Est = -1.21, *SE* = .299, *z* = -4.04, *p* <. 001; model without overly influential participants: group Est = -1.35, *SE* = .925, z = -4.57, *p* < .001), however the interaction effect of age and culture was no longer significant (model including all participants: interaction Est = .036, *SE* = .013, *z* = 2.88, *p* = .004; model without overly influential participants: interaction Est = .015, *SE* = .014, *z* = 1.07, *p* = .284): there was no longer an age effect on the chances of having a child in proximity in the UK when these participants were excluded. A conservative interpretation of these results would be that there is a main effect of group of whether a child is in proximity to infants; there is no age effect in Ugandan participants; and there is also no true age effect in UK participants.

#### Infant social experience—Infant social activities

**Fig 3:**
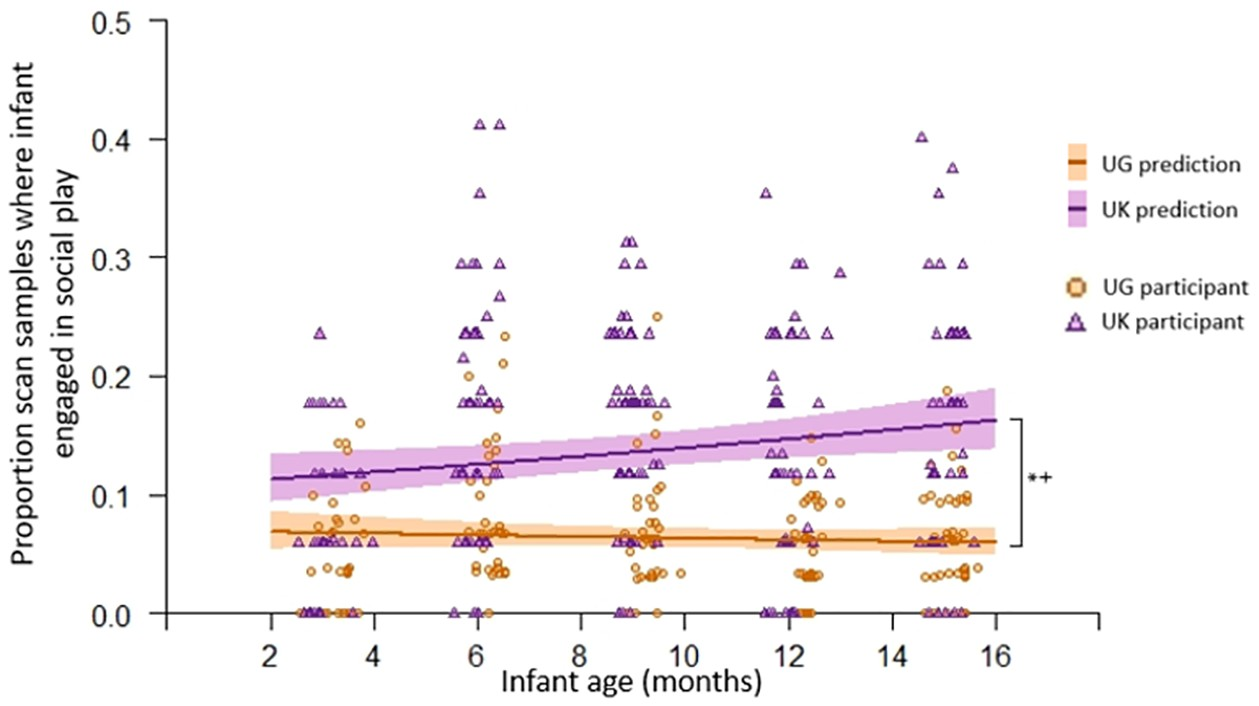
Proportion of scan samples where infant engaged in social play (circles/triangles) and the expected probabilities of this given GLMM results (lines) as they age. Note: UG = Ugandan. Shading indicates 95% confidence intervals. The number of scan samples in the UK where infant activity was known was more standard across participants, hence clusters are more commonly seen in the observed values for individual UK participants. * indicates significant group effect at *p* < .05 level, + indicates significant interaction effect at *p* < .05 level.

There was an interaction between group and infant age for how likely infants were to be engaged in social play (Likelihood Ratio test: *χ*^2^_(2)_ = 69.2, *p <* .001; Age*Group: Est = .041, *SE* = .018, *z* = 2.35, *p* = .019; Table N in S3 Results of S1 File). As Fig 3 suggests, post-hoc models confirm UK participants were more likely to display social play as they age, but there was no effect of age on Ugandan participants’ frequency of social play (post-hoc models: UK age *Est =* .*030*, *SE* = .011, *z* = 2.64, *p* = .008; UG age *Est = -*.*012*, *SE* = .013, *z* = -.091, *p* = .361). Overall, infant social play was more common in the UK than Uganda.

As infants aged, the chances they engaged in a social activity reduced (Likelihood ratio test: *χ*^2^_(2)_ = 39.7, *p <* .001; Age: Est = -.034, *SE* = .008, *z* = -4.20, *p* < .001; Table N and Figure H in S3 Results of S1 File). There was no group difference between UK and Ugandan infants regarding their likelihood of engaging in social activities (including play) and there was no significant interaction between group and infant age (Table N in S3 Results of S1 File).

#### Infant social experience—Infant interaction partners

Descriptively, mothers were the primary interaction partner for all infants, except one UK participant who had an equal number of interactions with their mother and grandmother (social interactions with mother: UK *M* = 79%, *SD* = 13.6; Uganda *M* = 83%, *SD* = 10.4). Father-infant interactions were recorded at least once for 48% of UK participants, and for 23% of Ugandan infants. Fathers made up *M =* 30% (*SD* = 41.6) of non-mother social interaction partners in the UK, and *M =* 4% (*SD* = 16.7) in Uganda.

**Fig 4:**
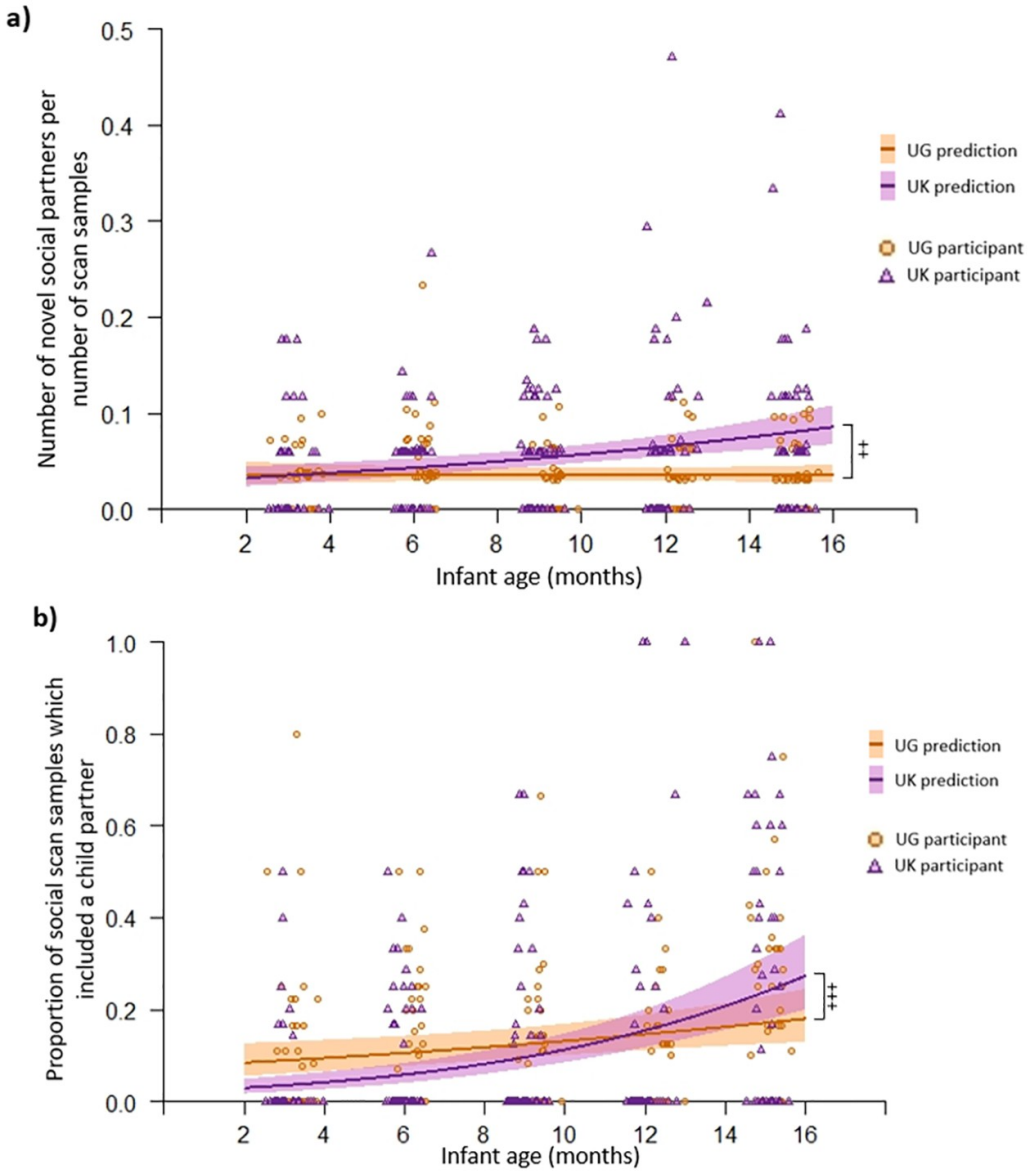
Graphs indicating significant effects found in infant social partner GLMMS. (a) Actual ratio of number of social partners to number of scan samples (circles/triangles) and the expected probabilities of this given GLMM results (lines) as they age. (b) actual proportion of social activity scan samples where social partner included a child social partner (circles/triangles) and the expected probabilities of this given GLMM results (lines) as they age. Note: UG = Ugandan. Shading around the lines show 95% confidence intervals. The number of scan samples in the UK where infant activity was known was more standard across participants, hence why clusters are more commonly seen in the observed values for individual UK participants. ++ indicates significant interaction effect at *p* < .01 level, +++ indicates significant interaction effect at *p* < .001 level.

A GLMM revealed a significant interaction between group and age for the likelihood infants had a novel interaction partner (Likelihood ratio test: *χ*^2^_(2)_ = 31.3, *p <* .001; Group*Age: Est = .075, *SE* = .024, *z* = 3.06, *p* = .002; Fig 4a; Table O in S3 Results of S1 File). Post-hoc models confirmed the likelihood of infants having novel interaction partners did not change with age in Uganda (post-hoc model: Uganda age Est = -.002., *SE* = .017, z = -.119, *p* = .905), but chances of having a novel interaction partner increased with age in the UK (post-hoc model: UK age Est = .075, *SE* = .017, z = 4.40, *p* < .001), meaning that by 15 months, UK infants were more likely to have more novel interaction partners than Ugandan infants.

There was an interaction between group and infant age on the probability that infants’ social partners included a non-mother adult (Likelihood ratio test: *χ*^2^_(2)_ = 35.5, *p <* .001; Group*Age: Est = .129, *SE* = .042, *z* = 3.06, *p* = .002; Table O in S3 Results of S1 File). Post-hoc models indicate that the likelihood of infant interaction partners to be a non-mother adult did not change with age in Uganda (post-hoc model: Uganda age Est = -.065., *SE* = .036, z = -1.84, *p* = .065; Bonferroni corrected alpha level = .025), however UK infants’ interaction partners were significantly more likely to be a non-mother adult as they aged (UK age Est = .064, *SE* = .023, z = 2.82, *p* = .005). Three UK participants and three Ugandan participants were deemed overly influential in the main model. These participants all had a high proportion of their social interactions with non-mother adults, and this increased over time. When the model was re-run without these participants, the interaction was not significant (interaction Est = .088, *SE* = .046, *z* = 1.90, *p* = .058): the increased chances of having a non-mother adult partner with age in the UK was less strong when the overly influential participants were not included. This indicates this effect is unstable, and should be interpreted with caution.

There was an interaction between group and infant age on how likely infants’ interaction partners were to include a child (Likelihood ratio test: *χ*^2^_(2)_ = 61.8, *p <* .001; Group*Age: Est = .119, *SE* = .034, z = 3.51, *p <* .001; Table O in S3 Results of S1 File). Fig 4b illustrates that engaging in social activities with a child partner was more common in Uganda at young ages, but more common in the UK at older ages. Post-hoc tests show both UK and Ugandan infants were more likely to have a child interaction partner as they aged, however this effect was stronger in the UK sample (post-hoc tests: UK age Est = .191, SE = .027, *z* = 6.94, *p* < .001; Uganda age Est = .057, *SE* = .021, *z* = 2.67, *p* = .008).

#### Proximity between infant and caregivers—Mother proximity during the day

**Fig 5:**
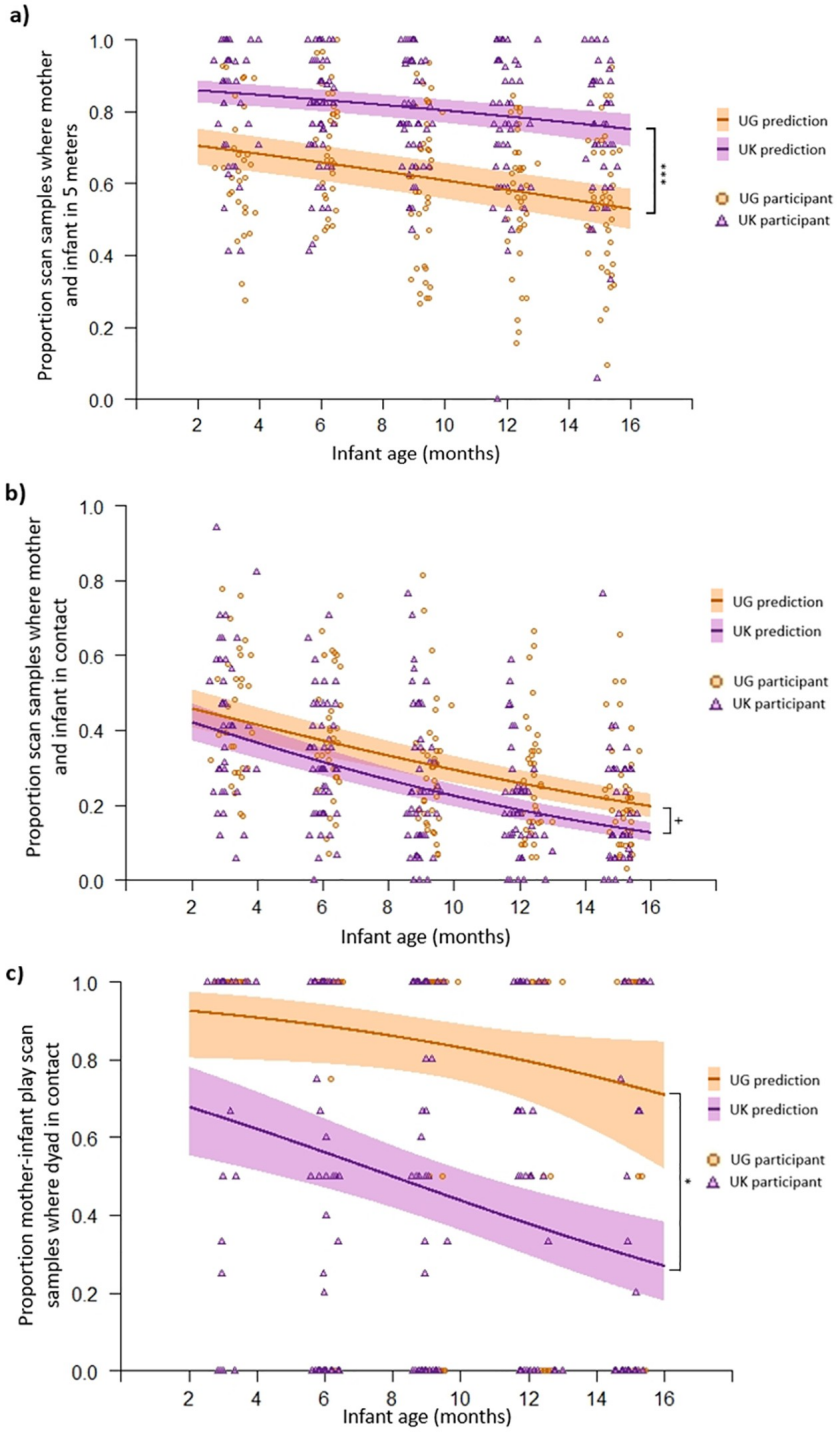
Graphs indicating significant effects found in mother-infant proximity, contact, and contact during play GLMMs. (a) Actual proportion of scan samples where mother-infant dyads were within five metres of each other (circles/triangles) and the expected probabilities of this given GLMM results (lines) as they age. (b) Actual proportion of scan samples where mother-infant dyads were in physical contact with each other (circles/triangles) and the expected probabilities of this given GLMM results (lines) as they age. (c) Actual proportion of scan samples where mother-infant dyads were in contact during play (circles/triangles) and the expected probabilities of this given GLMM results (lines) as they age. Note: UG = Ugandan. Shading around the lines show 95% confidence intervals. * indicates significant main effect of group at p < .05 level. *** indicates significant main effect of group at *p* < .001 level. + indicates significant interaction effect at *p* < .05 level.

Considering all occasions, mothers and infants in the UK were more likely to be within five metres of one another than dyads in Uganda (Likelihood ratio test: *χ*^2^_(2)_ = 117, *p <* .001; Group: Est = .926, *SE* = .192, z = 4.82, *p <* .001; Fig 5a; Table P in S3 Results of S1 File). Dyads in both groups were less likely to be within five metres of one another as the infant aged (Age: Est = -.054, *SE* = .007, z = -7.31, *p <* .001). There was no interaction between group and age of infant. To establish whether this was because caregiving is more shared in Uganda, or whether this is a feature of mothers’ caregiving style, we ran the same GLMM reduced to only scan samples where the mother was the caregiver and the same group and age effects were found (Table Q and Figure I in S3 Results of S1 File). This indicated that less time spent in five meter proximity between Ugandan mothers and infants, compared to dyads in the UK was a feature of mothers’ caregiving style.

Considering all occasions, there was an interaction between group and age on how likely infants were to be in physical contact with their mothers (Likelihood ratio test: *χ*^2^_(2)_ = 291, *p <* .001; Group*Age: Est = -.027, *SE* = .012, z = 2.19, *p* = .029; Table P in S3 Results of S1 File). Fig 5b illustrates infants from the UK and Uganda had similar likelihoods of being in body contact with their mothers, and across both groups infants were less likely to be in contact with their mother as they got older, especially in the UK (post-hoc models: UG age Est = -.088, *SE* = .008, *z* = -11.7, *p* < .001; UK age Est = -.116, *SE* = .010, *z* = -11.9, *p* < .001). The same GLMM performed on scan samples, when considering only occasions when mother was carer, infants in both cultures were less likely to be in contact with their mother as they aged, but overall Ugandan infants were more likely to be in contact with their mothers than UK infants (Table Q and Figure I in S3 Results of S1 File).

UK infants were less likely to be in contact with their mothers during play than Ugandan infants (Likelihood ratio test: *χ*^2^_(2)_ = 55.9, *p <* .001; Group: Est = -1.75, *SE* = .733, *z* = -2.39, *p* = .017; Fig 5c; Table P in S3 Results of S1 File). Both UK and Ugandan infants were less likely to be in contact with their mother during play as they aged (Age: Est = -.116, *SE* = .059, z = -1.98, *p* = .047). There was no interaction between group and age on the likelihood of dyads being in contact during play.

#### Proximity at night—Infant sleeping arrangements

UK infants were less likely to share a bed and share a bedroom with somebody else at night than Ugandan infants: sleeping in their own room and bed was common for UK infants, however no Ugandan infants were ever reported to sleep in a room alone at night, and only one Ugandan infant (at two time-points) slept in a bed alone at night. Due to limited variance in the Ugandan sample, group effects were not tested inferentially, and only tested for age affects in the UK sample. In the UK, infants were less likely to share a bedroom as they aged (Likelihood ratio test: *χ*^2^_(2)_ = 59.4, *p <* .*001*; Age: Est = -.565, *SE* = .123, *z* = -4.60, *p <* .001; Table R and Figure J in S3 Results of S1 File).

### Are parental attitudes associated with observed or reported parenting behaviour?

#### Mother-infant proximity

A Kruskal-Wallis test indicated that UK and Ugandan mothers who showed high agreement with the statement “*infants should be in close proximity to their mothers*” conversely spent a lower proportion of their time within five metres of their infant (*n* = 56; median = 0.60; IQR = 0.50–0.80) than mothers who showed low agreement to the statement (*n* = 36; median = 0.76; IQR = 0.60–0.87; H_(1_ = 5.00, *p* = .025).

A Kruskal-Wallis test indicated that Ugandan mothers who showed high agreement to the statement *“infants should develop independence in the first 3 years of life*” spent a similar proportion of time within five metres of their infant (*n* = 30; median = 0.60; IQR = 0.50–0.64) to mothers who showed low agreement to the statement (*n* = 11; median = 0.50; IQR = 0.47–0.67; UG: H_(1)_ = .157, *p* = .692). The association between UK mothers’ attitudes towards this statement and their proximity with their infant was not examined since only 2/51 mothers showed low agreement with the statement.

#### Sleep location

For UK mothers, a Fisher’s exact test revealed an association between level of agreement with the statement that “*it is good for an infant to sleep alone*” and having an infant who slept in a room on their own (*p* < .001; 23/27 infants of high agreement mothers slept alone and 13/22 infants of low agreement mothers slept alone). All Ugandan infants shared a room at night, and 32/38 Ugandan mothers indicated low agreement that it was good for an infant to sleep alone, indicating good correspondence between attitude and behaviour for Ugandan participants.

#### Mother-infant time

A Kruskal-Wallis test indicated there was no difference in the proportion of time UK mothers spent caring for or socialising exclusively with her infant between mothers who showed high or low agreement with the statement “*It is important to devote a lot of time exclusively to the baby*” (H_(1)_ = .992, *p* = .319). This statement could not be adequately translated in Uganda, hence this analysis focusses exclusively on UK mothers.

## Discussion

In line with predictions, UK mothers in the present study held less relational and more autonomous views of parenting practices and socialisation goals than Ugandan mothers. Our first analyses confirmed UK and Ugandan participants formed two distinct groups based on mothering attitudes and infant early life environment, with no evidence of reliable further subgroups within the Ugandan sample, suggesting at least on these measures the Ugandan ethnolinguistic groups pattern together. Although there was considerable individual variation in attitudes regarding parenting practices and socialisation goals, overall we still found significant differences in attitudes between UK and Ugandan mothers in line with some previous findings. Specifically, that WEIRD mothers tend to hold more autonomous views and mothers from rural subsistence farming communities tend to hold more relational views [4, 68]. Our measures may have underestimated the difference between the groups, as two questions regarding autonomous values and the concepts of ‘self-esteem’ and ‘sense of self’ could not be successfully translated in Uganda, so were not included in the questionnaire. Given the strong relationship between culture and language [82–84], it is possible that difficulties in translating these concepts into single statements reflects the fact that autonomous concepts are not as relevant in this society (cf., [85–87]). If we had been able to find a way to express these concepts, it is possible we would have seen even greater differentiation between groups in regards to their socialisation attitudes.

Parental attitudes are often assumed to influence parenting behaviour and interactions with infants, thereby shaping the early life experiences of infants. However, few previous studies have taken an integrative approach to measure multiple aspects of infant early life environment as well as experiences spanning different stages of early development. In this study, we were able to rigorously characterise multiple aspects of early life at multiple time points from 3–15 months in families sampled from the UK and Uganda, and crucially test behavioural predictions of the relational-autonomous parenting models. Whereas some predictions were clearly supported by our behavioural data (e.g., more distributed infant care in Uganda), other predictions were only partially supported by some behavioural measures (e.g., mother-infant proximity), and other predictions were not supported at all (e.g., number of infant social interaction partners) (see also [20, 21, 66]).

In line with the more distributed child care expected in relational parenting [15], we found infants in the UK sample had significantly less caregivers than infants in the Ugandan sample, even though the mother was the primary caregiver in both groups. UK infants were significantly less likely to have a non-mother adult caregiver than Ugandan infants. Although a similar percentage of UK and Ugandan participants were cared for at least once by their fathers, fathers acted as caregivers for their infants more frequently in the UK than Uganda. Arguably the most striking difference between the two groups was that child caregivers were never witnessed in the UK context but were common for Ugandan infants. Child caregivers were observed at least once in each of the Ugandan families and at least once in ~80% of Full-Day follows, indicating this may also be a key cultural difference. Relational maternal attitudes may be only one factor of relevance here. For example, in Uganda, there were more households with older children (67% with children aged 6–10, and 56% with children aged 11–16; compared to 9% and 4% in the UK respectively). This difference in household structure may have contributed towards lower rates of child caregivers in the UK. In both samples there were households with children other than the study infant under 5 years (47% in UK, and 66% in Uganda). In the UK these infants may have been deemed not old enough to look after a young infant, but 66% of Ugandan study infants were reported to have a carer under 5 at least once. So both practical considerations and cultural norms about the responsibility given to young children likely contributed to the differences seen.

There were also differences between children in the two cultures in reaching physical milestones, with Ugandan infants able to sit, crawl, and walk earlier than the UK infants. As physical stimulation of infants by caregivers is emphasised in rural agrarian societies and interdependent cultural models [4], the earlier attainment of physical milestones by Ugandan infants could be due to this. For example, Ugandan mothers often lift their infants by a single upper arm, whereas UK mothers tend to lift symmetrically often whilst supporting the head and neck. Future studies should seek to systematically document how physical stimulation in play, as well as lifting, holding and carrying postures differ between groups to better understand what drives earlier achievement of physical milestones (see also [13]).

Whilst earlier infant physical development and distributed caregiving in the Ugandan sample fully supported the predictions of interdependent-independent cultural models and relational-autonomous models of parenting, other aspects of infant life did not show such clear patterns. We predicted that the value placed on infant independence in more autonomous parenting would mean UK mothers maintained less proximity with their infants than Ugandan mothers. We found mixed support for this. UK infants in this study were less proximate to their mothers at night, with the majority sleeping in their own bed and room once they were a bit older, while, Ugandan infants virtually all shared a bed and room across all ages we considered. Infant sleeping locations are likely also influenced by logistical factors, such as availability of space and technology (e.g., baby monitors that allow for remote monitoring of infants). Co-sleeping arrangements may also be impacted by breastfeeding practices, which may be linked to interdependent-independent attitudes or practical considerations. Few UK mothers breastfed as long as Ugandan mothers, perhaps because they can more easily access alternative sources of infant food. In contrast to sleeping arrangements, during the day the UK mothers were significantly more likely to be within five metres of their infant than the Ugandan mothers. This finding, in the opposite direction to predictions, may have resulted from differences in housing environments. Given the compound structure of Ugandan homes (open area of land with several small buildings) and the tendency for families to spend a lot of time outside, Ugandan mothers may have been able to effectively observe and interact with their infants from a greater distance, whereas UK mothers, who spent more time inside their homes were more likely to be in the same room as their infant (generally less than five metres).

A natural extension of proximity between infant and mother is body contact, which previous studies have noted to be more prevalent in relational parenting models [4, 9, 10, 15–18]. Here our data concurs. Infant-mother body contact decreased with infant age, but more steeply in the UK sample, meaning Ugandan children had more body contact with their mothers compared to the UK infants and this was increasingly pronounced with age. Again, this pattern may be linked to breastfeeding practices, as more Ugandan mothers breastfed until older ages. We also examined body contact as a function of the time spent with the mother as caregiver. Whilst body contact still declined as the infant aged, the interaction between group and age became non-significant, and instead we saw a clear effect of group—with Ugandan mother-infants being in more body contact than UK mother-infants over all ages. We found an equally clear and consistent effect of group on body contact within social play contexts, with Ugandan mother-infants in the sample engaging in significantly more body contact during social play than UK mother-infant dyads at all ages. Previous literature has largely focussed on characterising the degree of body contact within social play contexts at a single time point [9, 10, 16–18]. Our study extends these previous findings, showing that Ugandan mothers were more likely than UK mothers to be in body contact with their infants whilst playing with them in the range of 3–15 months of age, and critically these differences generalise to non-play contexts when the mother remained the caregiver for her infant.

So far, the aspects of infant early life we have considered either aligned with the relational-autonomous predictions or can potentially be explained by other environmental factors. However, we also identified some aspects of the social environment where predictions were not well supported. We predicted the greater importance placed on social context in relational parenting would mean Ugandan infants had more people in proximity and a greater number of social interaction partners, but we found little empirical support for either. We found there was no difference in the number of people within five metres of the infants in the UK and Uganda, which was particularly unexpected given the larger household sizes in the Ugandan sample. Although the Ugandan infants in this study had a relatively stable number of potential interaction partners in close proximity, the participating UK infants may have had more variation in experiences, with fewer people in proximity within the family home being offset by relatively frequent outings to public places or group activities (e.g., parks, play groups) where high numbers of individuals may have been within five metres. In line with this, infants in both groups were equally likely to have a non-mother adult within five metres, indicating their potential opportunity for interacting with adult partners was similar. Interestingly, UK infants were significantly less likely to have another child within five metres of them than Ugandan children, indicating they have less opportunity for observation of and interaction with peers than their counterparts.

We considered people in five metres of the infant as potential interaction partners, yet when we examined who actually interacted with infants, the prediction that infants in more relational societies would have a greater number of social interaction partners was also not supported. The number of novel interaction partners for infants stayed low and stable across ages in Uganda, but increased with age in the UK infants; so older infants from the UK had more novel interaction partners than infants from Uganda. However, UK and Ugandan infants were equally likely to have a non-mother adult as a social activity partner, and although Ugandan infants were more likely to interact with another child at younger ages, UK infants were more likely to engage in social activities with a child partner later. These findings suggest that once they reach the end of their first year, the UK infants are socially interacting with a higher number of different individuals and are more likely to engage in social activities with another child, compared to the infants from rural Uganda. This is particularly surprising given the UK children in this study were less likely to be in close proximity with another child overall; and yet they had more social interactions.

Finally, we found the time infants spent engaging in social activities to be similar across groups, as predicted. However, when we isolated social play as a key type of social activity [44, 88–91], we found an unexpected interaction between age and group, with the Ugandan infants maintaining stable low levels of social play across age, and the UK infants showing a significant increase with age. Social play provides important opportunities for facilitating the development of communication and linguistic skills, joint attention, and joint action [88–91], so whether these group differences in frequency of social play are maintained at later ages, and whether this differential engagement leads to differences in linguistic or social cognition skills requires future investigation [92]. Intriguingly, recent studies suggest linguistic development proceeds at similar rates, regardless of cultural differences in interactional styles [93].

It is important to consider some methodological limitations of our study before reaching final conclusions. Some differences we detected between cultures could have been an artefact of differences in methodology. Most notably, all Ugandan mothers were directly observed during Full-Day Follows, but all UK mothers opted to receive receiving phone calls every 30 minutes to report their activities, rather than take part in direct observation. Although both methods could be considered intrusive and social desirability could have influenced what mothers did or reported, it is difficult to estimate the degree or direction in which these factors may have influenced our results. In future, performing direct observation on families from both populations will be important to confirm the results we report here. Additionally, in terms of questionnaire delivery, UK mothers completed questionnaires with paper and pen, but low rates of literacy meant Ugandan mothers completed the questionnaire verbally. This may have made Ugandan mothers more susceptible to providing socially desirable answers. Nevertheless, we observed a good distribution of responses in the Ugandan population, indicating either social desirability did not overtly influence responses or there was no obvious ‘right’ answer for most questions. Lastly, familiarity with Likert scales was likely lower in Uganda than the UK at the start of our longitudinal project. To mitigate this, we completed a ‘Warm-up Questionnaire’ with mothers, so they could understand the structure of the response scale (see S1 Methods in S1 File). As the participants in this study were also part of a larger longitudinal project, the mothers in both samples were presented with other Likert scale questionnaires multiple times before the 11-month investigation, meaning all participants had experience with scales. Despite these measures, we still observed differences in how participants in UK and Uganda responded. It is still unclear whether differences in response styles (e.g., using extreme answers in Uganda) were due task familiarity with or cultural differences in expressing opinions. Importantly, however, we evaluated mothers’ attitudes towards socialisation goals in two different ways (Likert scale and forced-choice paradigm) and the results from both measures aligned, giving us confidence in the overall findings.

Taken together, our results show broad support for most predictions made by relational-autonomous parenting models. We did, however, identify a number of predictions that were not wholly supported, highlighting the importance of systematically measuring behaviour instead of simply extrapolating expected behaviour on the basis of parenting attitudes or broadly categorising participants. Our results not only identified clear differences in early life experience of rural Ugandan and UK infants, but they also demonstrated that many aspects change dynamically over time and these changes are not always uniform across groups. Focussing on a single time point would have generated potentially misleading findings, demonstrating the importance of sampling behaviour across ages using a longitudinal approach when trying to understand the context for infant development.

In addition to examining cultural differences, we also examined the relationship between reported attitudes and corresponding behaviour at an individual level. Individual views on whether an infant should sleep alone showed strong correspondence with reported behaviour, but we failed to find correspondence between behaviour and agreement with other questionnaire items. Specifically, physical proximity of the mother to her infant was not related to the extent to which she agreed with statements regarding the importance of the infant being in close proximity or developing independence. There was also no relationship between the proportion of time UK mothers dedicated to activities exclusively for their infant and their agreement with the statement that mothers should devote a lot of time exclusively to the baby. This lack of correspondence may be due to several factors. First, the wording of statements is open to interpretation, and diverse interpretations may have been at play. Second, it could be that individual questions are not reliable predictors of behaviour, but higher-level classifications of attitudes (e.g., relational-autonomous) are more predictive. Future research could address this by examining if individual variation within relational and autonomous parenting models predicts behaviour accurately.

In conclusion, we found that infants’ early life environment varies in many important ways in our two study groups in York, UK and the Nyabyeya parish, Uganda. Overall, the Ugandan mothers aligned with a more relational parenting model, and the UK mothers with a more autonomous parenting model. Although most behaviours fell in line with predictions of relational-autonomous parenting models, we did find some unexpected results which did not align well with these models. This highlights the importance of measuring behaviour, rather than extrapolating expected behaviour based on attitudes alone. In contrast to the attested cultural differences, individual variation in specific parenting attitudes was generally a poor predictor of behaviour. The Ugandan and UK infants in this study experienced many differences in their social environment in their first 15 months of life, highlighting the importance of looking beyond WEIRD children to document and understand the impact of parenting attitudes and social environments on infant development in diverse cultural settings.

## Supporting information

S1 File (DOCX)
