# Supplementary material for: Maternal attitudes and behaviours differentially shape infant early life experience: A cross cultural study
Source: PLoS One. 2022 Dec 21;17(12):e0278378. doi: 10.1371/journal.pone.0278378 (PMC9770339; doi:10.1371/journal.pone.0278378)
Supplement: S1 File — (DOCX) [file pone.0278378.s001.docx]

# Supplementary Information

## S1 Methods. Warm up Questionnaire

A ‘Warm-up Questionnaire’ was presented to UK mothers at 3 months and Ugandan mothers at 3 and 11 months to familiarise participants with the concept of 5-point Likert scales. The purpose of the questionnaire was to try to demonstrate to participants that it is possible to have different levels of agreement with a statement, and ‘warm them up’ to the use of the Likert scale used in subsequent questionnaires, thus responses were not analysed in this study. Local people were consulted on the cultural appropriateness of the statements included in the Warm-up questionnaire.

## S2 Methods. Full-Day Follow data collection appointment organisation

Mothers were encouraged to select a day where they or another family member was caring for the child, and so no Full-Day Follows were conducted while the infant was in a childcare facility. Although many UK mothers had professions, many were on maternity leave or working part-time during the study – thus the main caregiver in Full-Day Follows was usually the mother, father, or grandparent. Although mothers were asked not to change their plans to accommodate data collection, we noticed a pattern in days chosen for Full-Day Follow appointments in both UK and Ugandan samples. As implied above, UK mothers usually avoided days when they were working, and Ugandan mothers usually avoided days where they had planned to visit a market or had planned to collect firewood.

## S3 Methods. Explanation of behavioural categories extracted from the Full-Day Follow data

We categorised mother and infant’s activity during full day follows according to the categories and definitions provided in Table A. Carers were defined as the person responsible for the child. To help operationalise this we tried to determine who the mother would expect to respond if the child became distressed. The infant’s proximity to their mother was recorded using the categories explained in Table B. The number, and where possible the age category (child or adult) of people within 5 meters of the infant and within the infant’s sight (whichever was smaller) was recorded as a measure of their immediate social environment.

**Table A**. Definition of activities for mothers and infants, and whether a social partner should be recorded or not.

| ***Activity name*** | ***Explanation*** | ***Social partner?*** |
| --- | --- | --- |
| Feeding self | Eating something (i.e. putting food in your own mouth) | No |
| Feeding other | Mostly an activity for the mothers or carers: Feeding the infant with a bottle or giving them solid food | Yes |
| Nursing | Breastfeeding an infant | Yes |
| Being fed | Mostly an activity for the infants: includes being breastfed, being bottle fed, and being fed solid food | Yes |
| Infant care | Activity for the mothers or carers only: includes brushing hair, wiping face, washing hands, changing nappies, dressing the infant (Also applied to other children the mother or carer was looking after.) | Yes |
| Being cared for | The infant receiving the above mentioned care | Yes |
| Care for self | Dressing oneself, going to the bathroom etc. | No |
| Chatting | Being involved in an ongoing conversation/social interaction where chatting is the focus.  Specifications for the data collectors:   - If the mother is doing chores, eating etc. whilst also talking to someone, you should still code the chore or eating. - However, chatting is more active than resting. So we will code “chatting” when the mother is resting but engaging in listening to or talking to another as part of a social interaction. - Chatting is not coded for single vocalisations like a brief exchange, greeting someone or saying something one off. In these situations, the dominant context should be coded. - Chatting between mother and older infants also counted | Yes |
| Play solo | Playing alone without an object (e.g. running around by yourself) | No |
| Play solo object | Playing alone with an object (e.g. playing with a toy car) | No |
| Play social | Playing with another person without objects (e.g. hide and seek) | Yes |
| Play social object | Playing with another person with objects (e.g. two children playing with a ball, mother reading book to infant) | Yes |
| Resting | Includes activities like sitting resting, interacting with mobile phone (unless on a phone call), watching tv, reading a book to self, no social interactions.  Also coded when the infant was simply being held. | No |
| Sleeping | Being asleep | No |
| Active Travelling | Moving outside the house to achieve a goal (e.g. going to the shops, walking to the doctor’s office, the hairdresser).  The individual is exerting energy while travelling: e.g. walking or riding a bike. This category did not include moving around inside the home. | No |
| Passive Travelling | Moving outside the home to achieve a goal (e.g. driving to the shops, taking the bus to the doctor’s office, the hairdresser). The individual is not physically active while travelling: e.g. going by bus, driving in a car, being pushed in a pushchair, being in a car seat, being carried in a sling.  This category did not include moving around inside the home. | No |
| Exploring | Moving around within the local area (e.g. the home, the room, the waiting room at the doctor’s, the park), that is NOT travel or play.  The movement was not essential (no end goal like travelling to the shops), but it could be movement towards an object (e.g. an infant crawling towards a toy). Includes movements like crawling, bum shuffling, walking, sofa cruising. For adults it also included running in the park to self-exercise or walking around in the garden for relaxation. | No |
| Distress | Being distressed, e.g. infant is crying | No |
| Comfort other | Comforting someone else, e.g. holding them in arms plus doing something reassuring like rocking, shushing etc. | Yes |
| Household chores | Mostly an activity for the mothers or carers: Doing chores like preparing meals, sweeping, washing up…  Only considered a household chore for infant if they are helping somebody else, i.e. another older individual is doing this at the same time as them, such as infant trying to help mother clean clothes, or helping mother wipe a table. | Mother: no  Infant: yes |
| Work | Refers to jobs for bringing in resources. Mother working with no potential for infant to be involved, e.g. digging fields, laptop, phone calls. | No |
| Essential Shopping | Buying essential items (e.g., food for the family or medication) | Mother: no  Infant: yes |
| Leisure Shopping | Buying less essential items (e.g., shirts)  NB: Online leisure shopping was coded as resting | Mother: no  Infant: yes |
| Other | Other rare context not included in the above list, or don’t know how to categorise. Include description of activity in comments. |  |

**Table B.** Definitions of categories used to characterise the body contact or distance between the infant and mother during full day follows

| ***Category*** | ***Explanation*** |
| --- | --- |
| Ventral | The infant is carried on the front of the mother’s body, for example when the mother is holding the infant in her arms or when the infant is carried in a sling on the mother’s chest |
| Dorsal | The infant is carried on the back of the mother’s body, for example when the infant is in a sling on the mother’s back |
| Body contact | Any body contact other than being carried ventrally or dorsally, for example infant on the mother’s lap, sitting next to her touching |
| Distance in meters | If there was no body contact at all between mother and infant, but the mother was in sight of the child, the distance between mother and infant that applied was selected (0 to 1, 1-2meters, 2-5meters, 5-10 meters, 10m+).  The mother-infant distance was coded as 10m+ if the mother was in a different room or area of the compound/house that was out of sight of the infant, even if the actual distance was less than 10m. |

## S4 Methods. Full-Day Follow data collection sheets

Figure A and B show the data sheets for Full-Day Follow data collection.

**Figure A. data collection sheet for UK Full-Day Follows**

| 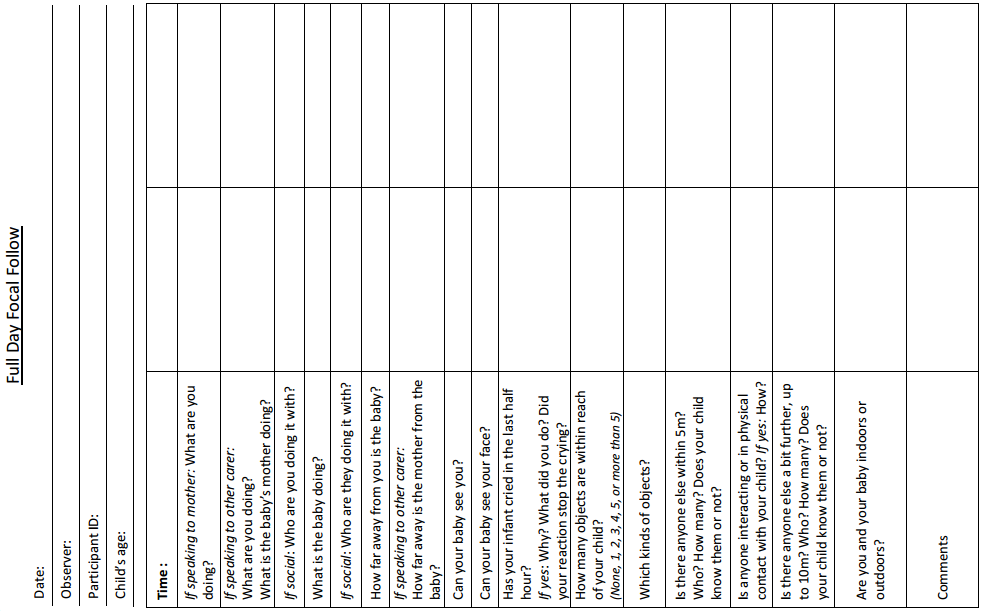 |
| --- |

**Figure B. data collection sheet for Ugandan Full-Day Follows**

| 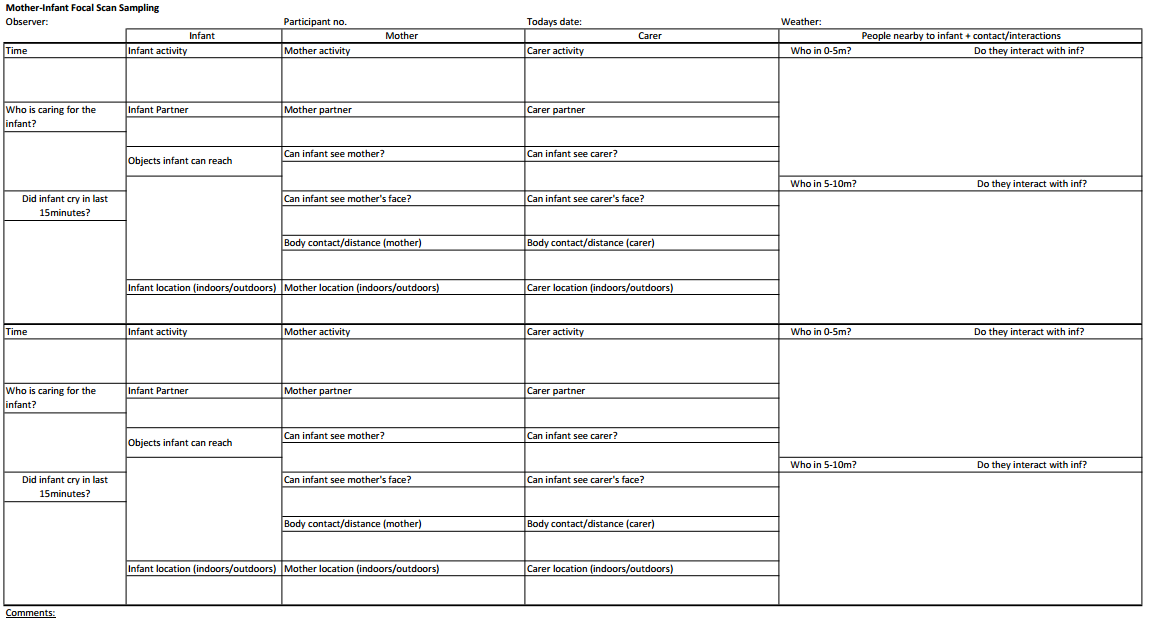 |
| --- |

## S5 Methods. Extraction of importance attributed to each socialisation goal

In addition to the calculation of the importance individual mothers attributed to relational vs autonomous goals (main manuscript), we also performed an item-level analysis: we calculated the relative score of importance for each of the six socialisation goal items that were used in the forced-choice task: For each goal, we took the average of the scores that all mothers in each group had given it across the three pairwise comparisons that goal had been part of. A high positive relative score of importance for an item would mean that many mothers chose this item as important no matter which item it was compared with. A high negative relative of importance score for an item would indicate that many mothers decided against this item regardless of which other item it was compared with. A relative importance score close to zero could indicate one of two things: it could either mean that some mothers chose this item and some mothers did not choose it. Alternatively, it could mean that this item was only chosen when compared to some items but was not chosen when compared to other items.

## S6 Methods. Principal Component analysis

A cluster analysis was conducted to determine if early life environment for infants: a) fell into two distinct groups (dyads from the UK and Uganda), and b) in Uganda fell into distinct groups based on ethnolinguistic group. To provide a holistic picture of infant early life environment, we included variables concerning mothers’ attitude towards infant independence and social environment, infant attainment of physical milestones and environmental exploration, and infant social environment and social interactions (see Table C). As we were interested if the overall pattern across features of infant early life environment created clusters of participants, prior to the cluster analysis, a principal component analysis (PCA) was conducted as a method of data reduction.

PCA analyses were run in *R* version 4.0.1 [1]. The *R* packages Corpcor [2], GPArotation [3], and psych [4] were used. Not all measures were available for all participants, thus, a Pearson’s correlation matrix was created which omitted individuals where a specific pairwise correlation was missing a value. Across the variables included in the PCA, 2.7% of total data was missing. The number of participants with missing data per variable included are detailed in Table D. The PCA was conducted on this correlation matrix.

**Table C. Details of measures included in PCA.** The domain that the variable aimed to measure is specified for each variable (Mothers attitudes, infant attainment of physical milestones, infant exploration of the environment, infant social environment, and infant social interactions). The average was taken across time-points (maximum five) available per participant using the method described in variable description.

| ***Specific measure (Domain grouping)*** | **Variable description** | **Data source*** | **Variable structure** | **Inclusion Criteria** |  |
| --- | --- | --- | --- | --- | --- |
| Parenting practices attitude *(Mothers’ attitude towards infant independence and social environment)* | Difference score calculated from Parenting Questionnaire answers | Parent. Q. | Numeric: difference score -4 to 4 | All available included |  |
| Socialisation goals attitude *(Mothers’ attitude towards infant independence and social environment)* | Difference score calculated from Socialisation Goals Questionnaire answers | Soc. Goals Q. | Numeric: difference score -4 to 4 | All available included |  |
| Sit *(Attainment of physical milestones)* | Time-point at which infant was first reported as being able to sit unsupported | Devo. Q. | Ordinal: 3, 6, 9, 12, 15months or not yet | All available included |  |
| Crawl *(Attainment of physical milestones)* | Time-point at which infant was first reported as being able to crawl | Devo. Q. | Ordinal: 3, 6, 9, 12, 15months or not yet | All available included |  |
| Walk *(Attainment of physical milestones)* | Time-point at which infant was first reported as being able to walk unsupported | Devo. Q. | Ordinal: 3, 6, 9, 12, 15months or not yet | All available included |  |
| Amount of Environment Exploration | Mean % of scan samples in a FDF across time-points where infant activity is solo-play or explore | FDF | Numeric: Mean % scans per time-point (maximum 5) | All available included |  |
| Number of Carers *(Social environment)* | Mean number of non-mother carers during a FDF across time-points | FDF | Numeric: Mean of count values per time-point (maximum 5) | Minimum of at least 11 scans per time-point |  |
| Adult Carer *(Social environment)* | Proportion time-points where a non-mother adult carer was present during the FDF | FDF | Numeric: Proportion out of maximum 5 time-points | Minimum of more than 10 scans per time-point |  |
| Child Carer *(Social environment)* | Proportion of time-points where a child carer was present during the FDF | FDF | Numeric: Proportion out of maximum 5 time-points | Minimum of more than 10 scans per time-point |  |
| Proximity with Mother *(Social environment)* | Mean % of scan samples in a FDF across time-points where dyad are within 5meters of one another | FDF | Numeric: Mean % scans per time-point (maximum 5) | All available included |  |
| Number of people in Proximity *(Social environment)* | Median number of non-mothers in proximity of the infant across scan samples at all time-points | FDF | Numeric: Median** number of people in proximity (pooled time-points***) | All available included |  |
| Adults in Proximity *(Social environment)* | Mean % of scan samples in a FDF across time-points where there is a non-mother adult within 5meters of the infant | FDF | Numeric: Mean % scans per time-point (maximum 5) | All available included |  |
| Children in Proximity *(Social environment)* | Mean % of scan samples in a FDF across time-points where there is a child within 5meters of the infant | FDF | Numeric: Mean % scans per time-point (maximum 5) | All available included |  |
| Shared Bedroom *(Social environment)* | Proportion time-points where infants were reported to share a bedroom with somebody else at night | BG. Q. | Numeric: Proportion out of maximum 5 time-points | All available included |  |
| Shared Bed *(Social environment)* | Proportion time-points where infants were reported to share a bed with somebody else at night | BG. Q. | Numeric: Proportion out of maximum 5 time-points | All available included |  |
| Contact with Mother *(social interactions)* | Mean % of scan samples in a FDF across time-points where dyad are in contact with one another | FDF | Numeric: Mean of % scans per time-point (maximum 5) | All available included |  |
| Mother Activities for Infant *(social interactions)* | Mean % of scan samples in a FDF across time-points where mothers activity is solely for the purpose of caring or interacting with the infant | FDF | Numeric: Mean of % scans per time-point (maximum 5) | All available included |  |
| Amount Social Play *(social interactions)* | Mean % of scan samples in a FDF across time-points where infant activity is social play | FDF | Numeric: Mean of % scans per time-point (maximum 5) | All available included |  |
| Contact During Play *(social interactions)* | % of dyad play instances where mother and infant are in contact across scan samples at all time-points | FDF | Numeric: % of play activity scan samples in contact (pooled time-points***) | Minimum of 3 scan samples where activity is mother-infant play across time-points |  |
| Amount Social Activities *(social interactions)* | Mean % of scan samples in a FDF across time-points where infant activity is a social activity | FDF | Numeric: Mean of % scans per time-point (maximum 5) | All available included |  |
| Number of Social Partners *(social interactions)* | Mean proportion of scans with non-mother social partners out of total scans during a FDF, across timepoints | FDF | Numeric: Mean of proportion value | All available included |  |
| Adult Social Partners *(social interactions)* | % of social events across scan samples across time-points where partner is a non-mother adult | FDF | Numeric: % of scan samples (pooled time-points***) | Minimum of 3 scan samples where activity is social across time-points |  |
| Child Social Partners *(social interactions)* | % of social events across scan samples across time-points where partner is a child | FDF | Numeric: % of scan samples (pooled time-points***) | Minimum of 3 scan samples where activity is social across time-points |  |
| *Key: FDF=Full Day Follow; Devo. Q.=Developmental Questionnaire; BG. Q.=Background Questionnaire; Soc. Goals Q.=Socialisation Goals Questionnaire; Parent. Q.=Parenting Questionnaire  ** Median was taken as the value for the number of people in proximity to avoid occasions when mothers were in a crowd for one scan sample, which would not be representative of their full day, having a large effect on the mean for that participant.  ***Time-points were pooled for cases where the scan samples were subset into only infant social activity scan samples, due to the relatively infrequent nature of these social activity scans | | | | | |

**Table D. Details of missing data for PCA analyses.**

| ***Variable*** | **Count of participants with missing data (of 97)** |
| --- | --- |
| Parenting practices attitude | 5 |
| Socialisation goals attitude | 5 |
| Sit | 6 |
| Crawl | 8 |
| Walk | 14 |
| Amount of Environment Exploration | 0 |
| Number of Carers | 0 |
| Adult Carer | 0 |
| Child Carer | 0 |
| Proximity with Mother | 0 |
| Number of people in Proximity | 0 |
| Adults in Proximity | 0 |
| Children in Proximity | 0 |
| Shared Bedroom | 0 |
| Shared Bed | 0 |
| Contact with Mother | 0 |
| Mother Activities for Infant | 0 |
| Amount Social Play | 0 |
| Contact During Play | 21 |
| Amount Social Activities | 0 |
| Number of Social Partners | 0 |
| Adult Social Partners | 1 |
| Child Social Partners | 1 |

To determine which variables were appropriate for inclusion in the PCA, a Bartlett’s test run on the correlation matrix (ꭕ*^2^*_(253)_ = 1576, *p* < .001) indicated the matrix was not an identity matrix. No variables shared little or high variance with another so all variables were included for further analysis. Finally, a Kaiser-Meyer-Olkin (KMO) test indicated the overall Measure of Sampling Adequacy (MSA) was .73, and no individual MSAs were below .5. This indicates all variables were adequate for inclusion. Following this, a six Principal Component (PC) solution was deemed adequate since the first six components had eigenvalues above 1 (Kaisers criterion [5]), and the last ‘step’ in eigenvalues was between Component 6 and 7 (See Figure C). The PCA was then re-run with only these 6 components, following [6].

| 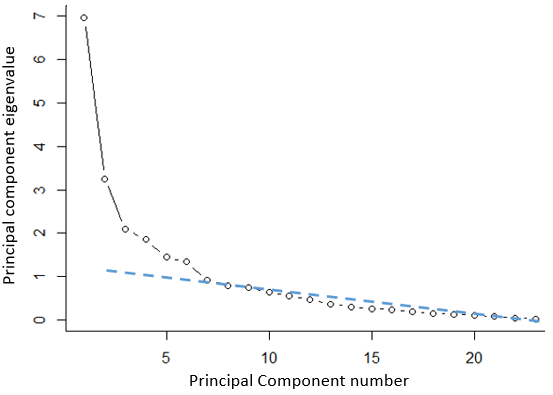 |
| --- |
| **Figure C: Graph of Principal Component (PC) eigenvalues.** PCs 1-6 six have eigenvalues greater than 1. You can also see the last ‘step’ in eigenvalue is between PC6 and PC7, as shown by the blue line following the trend from PCs7+ but not reaching PC6. |

Using the loadings from the extracted PCs, individual mother-infant dyad scores were calculated for each PC by summing the product of the *PC variable loading and the standardised variable value* for each dyad.

For example:

$${Participant}_{n} PC1 score=\left( {PC1 loading}_{variable1}\times{standardised {participant}_{n} score}_{variable1} \right)+\left( {PC1 loading}_{variable2}\times{standardised {participant}_{n} score}_{variable2} \right)+\left( {PC1 loading}_{variable3}\times{standardised {participant}_{n} score}_{variable3} \right)$$

$$\ldots up to+\left( {PC1 loading}_{variable 23}\times{standardised {participant}_{n} score}_{variable 23} \right)$$

Where *n* = participant dyad ID, and *variable* = a measure from *Table C.*

When calculating the PC score for a specific PC for any specific dyad, if the dyads did not have values for all variables, the mean value was used as the standardised participant score for that specific variable in calculations. The cluster analysis was then run on the participant dyad level scores for the 6 PCs.

## S7 Methods. Calculation of infant age in months

Infant age was entered into statistical models in months (calculated with the following formula:

$$Infant age \left( months \right)=\frac{Number of days old on day of data collection}{\frac{Number of days in a year}{Number of months in a year}}$$

## S8 Methods. Generalized Linear Mixed Models details

Generalized Linear Mixed Models (GLMMs) were run to examine whether variation in infants’ attainment of physical milestones; social environment; and social interactions (dependent variables), could be explained by cultural group, infant age, and the interaction between group and age (fixed factors). The full details of the factors, error structure, level of analysis (including N) and inclusion criteria for each model are provided in Table E.

**Table E. Details of measures included and error structure used for each GLMM.** FDF=Full-Day Follow; Devo Q=Developmental Questionnaire; BG Q=Background Questionnaire. ‘Scan samples’ are the individual data points collected during a FDF.

| **Dependent variable** | **Level of analysis** | **Dependent variables description (structure)** | **Model error structure** | **Fixed factors (Random factors) *** | **Inclusion criteria** |
| --- | --- | --- | --- | --- | --- |
| Sit *(attainment of physical milestones)*** | Questionnaire answer (n=260) | In the Devo Q was infant reported to be able to sit unsupported (Binary: Yes/No) | Binomial | Group + (Infant Age) + (Participant) | Only 3, 6, 9 month age-groupings |
| Crawl *(attainment of physical milestones)*** | Questionnaire answer (n=270) | In the Devo Q was infant reported to be able to crawl (Binary: Yes/No) | Binomial | Group + (Infant Age) + (Participant) | Only 6, 9, 12 month age-groupings |
| Walk *(attainment of physical milestones)*** | Questionnaire answer (n=262) | In the Devo Q was infant reported to be able to walk unsupported (Binary: Yes/No) | Binomial | Group + (Infant Age) + (Participant) | Only 9, 12, and 15 month age-groupings |
| Amount of environment exploration *(Non-social experience)* | Scan samples (n=9373) | In the scan sample was the infant’s activity exploratory (‘solo play’ or ‘explore’)? (Binary: Yes/No) | Binomial | Infant Age + Group + Infant Age*Group + (Participant) | All available included |
| Number of caregivers *(social environment)* | FDF (n=396) | Count of how many carers were present during the FDF (Numeric: Count) | Poisson*** | Infant Age + Group + Infant Age*Group + (Participant) | Minimum of more than 10 scan samples per FDF |
| Adult caregiver *(social environment)* | Scan samples (n=9322) | In the scan sample was a non-mother adult noted as the infants carer (Binary: Yes/No) | Binomial | Infant Age + Group + Infant Age*Group + (Participant) | Minimum of more than 10 scan samples per FDF |
| Child caregiver (UG only) *(social environment)* | Scan samples (n=5382) | In the scan sample was someone < 17 years noted as the infant’s carer? (Binary: Yes/No) | Binomial | Infant Age + (Participant) | Minimum of more than 10 scan samples per FDF. Only run for Ugandan sample due to no variation in UK (i.e. in 3944 scan samples, no UK participants ever had a child as their carer). |
| Number of people in 5 metres proximity *(social environment)* | Scan samples (n=9419) | In the scan sample how many non-mothers were in proximity (5 metres) of the infant (Numeric: Count) | Negative Binomial*** | Infant Age + Group + Infant Age*Group + (Participant) | All available included |
| Adults in 5 metres proximity *(social environment)* | Scan samples (n=9411) | In the scan sample, was there a non-mother adult within 5 metres of the infant? (Binary: Yes/No) | Binomial | Infant Age + Group + Infant Age*Group + (Participant) | All available included |
| Children in 5 metres proximity *(social environment)* | Scan samples (n=9405) | In the scan sample, was there somebody < 17 years within 5 metres of the infant? (Binary: Yes/No) | Binomial | Infant Age + Group + Infant Age*Group + (Participant) | All available included |
| Amount social play *(social interactions)* | Scan samples (n=9407) | In the scan sample was the infant’s activity social play? (Binary: Yes/No) | Binomial | Infant Age + Group + Infant Age*Group + (Participant) | All available included |
| Amount of social activities *(social interactions)* | Scan samples (n=9390) | In the scan sample was the infant’s activity a social activity? (Binary: Yes/No) | Binomial | Infant Age + Group + Infant Age*Group + (Participant) | All available included |
| Number of social partners *(social interactions)* | Scan sample (n=9308) | Did the infant have an interaction with a novel non-mother partner in the scan sample**** (Binary: Yes/No) | Binomial | Infant Age + Group + Infant Age*Group + (Participant) | All available included |
| Adult social partners *(social interactions)* | Scan samples where activity was social (n=2175) | In the scan sample did the infant’s social partners include a non-mother adult? (Binary: Yes/No) | Binomial | Infant Age + Group + Infant Age*Group + (Participant) | Minimum of 3 scan samples where activity is social across time-points |
| Child social partners *(social interactions)* | Scan samples where activity was social (n=2175) | In the scan sample did the infant’s social partners include someone < 17 years? (Binary: Yes/No) | Binomial | Infant Age + Group + Infant Age*Group + (Participant) | Minimum of 3 scan samples where activity is social across time-points |
| Mother in 5 metre proximity of infant – all occasions *(social environment)* | Scan samples (n=9384) | In the scan sample were the mother and infant within 5 metres of one another? (Binary: Yes/No) | Binomial | Infant Age + Group + Infant Age*Group + (Participant) | All available included |
| Mother in 5 metre proximity of infant - when mother was carer *(social environment* | Scan samples with mother carer (n=7944) | In the scan sample were the mother and infant in 5 metres of one another? (Binary: Yes/No) | Binomial | Infant Age + Group + Infant Age*Group + (Participant) | All available included |
| Mother-infant physical contact all occasions *(social interactions)* | Scan samples (n=9515) | In the scan sample were the mother and infant in physical contact with one another? (Binary: Yes/No) | Binomial | Infant Age + Group + Infant Age*Group + (Participant) | All available included |
| Mother-infant physical contact when mother was carer *(social interactions)* | Scan samples when mother was carer (n=7944) | In the scan sample were the mother and infant in physical contact with one another? (Binary: Yes/No) | Binomial | Infant Age + Group + Infant Age*Group + (Participant) | All available included |
| Contact with mother during play *(social interactions)* | Scan samples when infants activity was social play with mother (n=553) | When infant’s activity was social play with their mother in the scan sample, were mother and infant in physical contact with one another? (Binary: Yes/No) | Binomial | Infant Age + Group + Infant Age*Group + (Participant) | All available included |
| Shared bedroom (only UK) *(social environment)* | Questionnaire answer (n=240) | In the BG Q was infant reported to share a bedroom with someone at night? (Binary: Yes/No) | Binomial | Infant Age + (Participant) | Only UK sample due to no variation in Ugandan sample (i.e. of 200 UG questionnaires none reported the infant sleeping in a room alone). |
| * ***Fixed and random factor descriptions:*** *Infant age is a continuous variable in months (unless otherwise specified, it varied from 2 to 16 months). Group is a binary variable describing the cultural group (Uganda or UK). Participant is a nominal factor depicting each individual participant dyad.*  **** ***Infant age was included as a random factor in this model rather than as a fixed factor****. This wasa done because if the infant reached a physical milestone by one time-point, they would still have this capacity at later time-points. It is thus redundant to test whether there will be an increase with age because this is inherent with the definition of this variable. Thus the model asks the question, if a child was sampled from X age range, children from Uganda would be more likely to have reached X milestone.*  ******Model error structure decisions****: Count variables were checked for zero-inflation and overdispersion. For ‘Number of Carers’ the number of zeros was within expected range for a Poisson distribution and was not overdispersed, thus this model was run with Poisson error structure. The variable ‘Number of people in Proximity’ was zero inflated and overdispersed, thus a negative binomial error structure was used.*  *********Chance of having a novel non-mother social partner:*** *As a measure of how many individuals infants were likely to interact with in a day, each scan sample was scored as whether the infant was interacting with a novel individual. A novel individual was considered an individual that the infant had not yet interacted with in that Full-Day Follow, e.g. if an infant interacted with their sibling twice within the Full-Day Follow, only one of the two scan samples would be graded as having a novel social partner. If the infant interacted with two new individuals in one scan sample then the second individual was considered a novel individual in the next scan sample.* | | | | | |

## S9 Methods. Attitude-behaviour statistics

To test if maternal attitudes at 11 months were associated with observed or reported parenting behaviour at 12 months, we compared the behaviour of mothers who showed high and low agreement with the attitude question. Table F details the specific attitude questions and behavioural data that were used for these analyses

**Table F. Analyses run to investigate if parental attitudes were associated with observed or reported behaviour.** * Kruskal Wallis tests were chosen for Infant/mother proximity 1, Infant/mother proximity 2, and Mother Infant time, over Mann-Whitney U tests due to unbalanced variance across groups.

| **Topic** | **Attitude Question** | **Behaviour at 12 months (source)** | **Test type*** |
| --- | --- | --- | --- |
| Infant/mother proximity 1 | Parenting Q10. A baby should always be in close proximity with his/her mother, so that she can react immediately to his/her signals. | UG and UK pooled: Proportion of scans samples where mother and infant are in 5 metres of each other (Full-Day Follows) | Kruskal-Wallis test |
| Infant/mother proximity 2 | Socialisation goals - Infants should develop independence during the first 3 years of life. | UG only: Proportion of scan samples where mother and infant are in 5 metres of each other (Full-Day Follows) | Kruskal-Wallis test |
| Sleep location | Parenting Q7. It is good for a baby to sleep alone. | UK only: Does infant share bedroom (Background Questionnaire) | Fisher’s exact test |
| Mother Infant time | Parenting Q35. It is important to devote a lot of time exclusively to the baby. (This question was not asked in Uganda, so only analysing UK here) | UK only: Proportion of scan samples where mother activity is exclusively for the infant? (Full-Day Follow) | Kruskal-Wallis tests |

## S1 Results. Do British and Ugandan the participants form two distinct groups?

The six Principal Components (PCs) from the PCA explained 74% of variance in the data. See Table G for PC loadings and Table H for eigenvalues and variance explained by each component.

**Table G. Variable loadings on to Principal Components**. h2 (communality) is the proportion of common variance within a variable. Strongest loading per variable are indicated in bold. All loadings of .40 or above are indicated with *.

| **Variable** | **Standardized Loadings per Principal Component** | | | | | | **h2** | **KMO MSA** |
| --- | --- | --- | --- | --- | --- | --- | --- | --- |
|  | **PC1** | **PC2** | **PC3** | **PC4** | **PC5** | **PC6** |  |  |
| Parenting Attitude | **-0.86*** | 0.09 | -0.07 | -0.03 | -0.01 | 0.01 | 0.75 | .84 |
| Shared Bed | **0.86*** | -0.01 | 0.02 | -0.12 | -0.06 | 0.04 | 0.85 | .82 |
| Child Carer | **0.81*** | 0.01 | 0.01 | -0.15 | -0.08 | -0.21 | 0.84 | .82 |
| Number of Carers | **0.75*** | 0.34 | 0.01 | -0.08 | -0.11 | -0.23 | 0.86 | .79 |
| Shared Bedroom | **0.75*** | -0.08 | 0.19 | 0.02 | -0.08 | 0.14 | 0.71 | .85 |
| Contact During Play | **0.53*** | 0.04 | -0.09 | -0.05 | -0.23 | 0.15 | 0.44 | .80 |
| Sit | **-0.40*** | -0.05 | 0.39 | 0.10 | 0.15 | 0.08 | 0.35 | .84 |
| Adults in Proximity | -0.19 | **0.86*** | -0.17 | 0.01 | 0.00 | 0.01 | 0.79 | .65 |
| Adult Carer | 0.48***** | **0.74*** | -0.04 | -0.02 | -0.07 | -0.09 | 0.82 | .67 |
| Number of People in Proximity | 0.23 | **0.63*** | 0.50***** | -0.11 | 0.11 | 0.21 | 0.81 | .61 |
| Adult Social Partners | -0.27 | **0.61*** | -0.15 | 0.32 | 0.14 | -0.19 | 0.75 | .75 |
| Child Social Partners | -0.13 | -0.23 | **0.88*** | 0.04 | -0.12 | -0.22 | 0.86 | .50 |
| Children in Proximity | 0.32 | 0.05 | **0.8*** | -0.10 | 0.02 | 0.24 | 0.89 | .68 |
| Amount of Social Activity | 0.11 | -0.01 | -0.01 | **0.96*** | -0.10 | 0.07 | 0.87 | .66 |
| Amount of Social Play | -0.34 | 0.20 | 0.05 | **0.68*** | 0.09 | -0.12 | 0.78 | .80 |
| Mother activities for infant | 0.10 | -0.35 | -0.40***** | **0.49*** | 0.13 | 0.24 | 0.74 | .68 |
| Number of Social Partners | -0.18 | 0.24 | 0.39 | **0.41*** | 0.25 | -0.31 | 0.65 | .68 |
| Walk | 0.06 | -0.06 | -0.04 | -0.07 | **0.91*** | -0.13 | 0.8 | .55 |
| Crawl | 0.01 | 0.02 | -0.03 | -0.06 | **0.89*** | 0.11 | 0.79 | .59 |
| Amount of environment exploration | 0.21 | 0.05 | -0.13 | -0.32 | **-0.40*** | -0.11 | 0.46 | .82 |
| Mother in Proximity | -0.41***** | -0.1 | 0.02 | 0.05 | -0.02 | **0.72*** | 0.72 | .71 |
| Mother in contact | 0.54***** | -0.18 | -0.08 | 0.24 | -0.02 | **0.64*** | 0.83 | .51 |
| Socialisation Goals Attitude | -0.45***** | 0.27 | -0.08 | -0.07 | -0.02 | **0.63*** | 0.59 | .85 |

**Table H. Summary of the eigenvalues and % variance explained by the six principal components.**

| **Principal Component** | **% of Variance explained** | **Eigenvalues** |
| --- | --- | --- |
| PC1 | 24 | 5.43 |
| PC2 | 12 | 2.65 |
| PC3 | 10 | 2.31 |
| PC4 | 10 | 2.36 |
| PC5 | 10 | 2.27 |
| PC6 | 8 | 1.93 |

Figure D shows the complete separation for the two cluster solution for UK and Ugandan participants. Figure E and Table I show there is no clear clustering for the different Ugandan ethnolinguistic groups that for the three cluster solution for Ugandan participants. The membership of Alur and Lugbara participants to cluster 1 and 2 was no more likely than chance (*χ*^2^_(1)_ = .697, *p* = .404). Cluster 3 consisted of one Alur mother-infant dyad and one Banyuoro mother-infant dyad. Both Lendu participant dyads were assigned to cluster 1. For other ethnolinguistic groups where there were two or three mother-infant dyads (Banyuro and Kakwa) some dyads were assigned to cluster 1 and others to cluster 2.


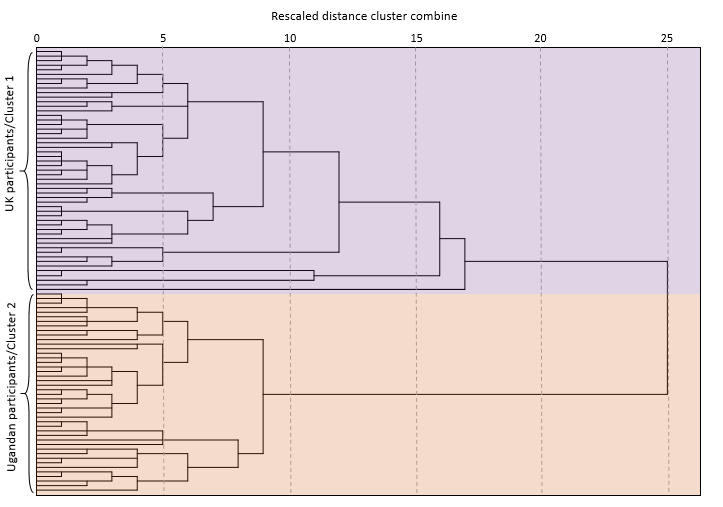


**Figure D. Dendrogram of hierarchical cluster analysis performed on all UK and Ugandan participants.** Note: each node on the left represents one mother-infant dyad.


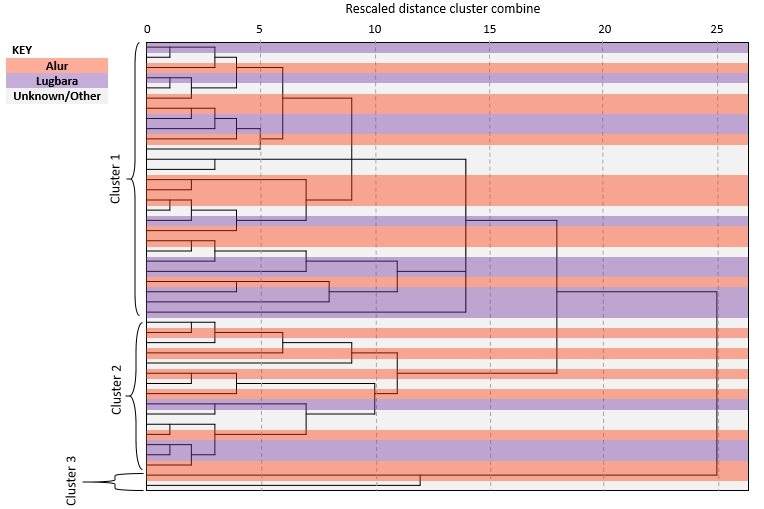


**Figure E. Dendrogram of hierarchical cluster analysis performed on only Ugandan participants.** Note: each node on the left represents one mother-infant dyad. Dyads are colour coded in line with ethnolinguistic group*.*

**Table I. Frequency table for number of mother-infant dyads assigned to each cluster.**

| **Ethnolinguistic group** | **Cluster membership (number assigned)** | | | **Total** | |
| --- | --- | --- | --- | --- | --- |
|  | **Cluster 1** | **Cluster 2** | **Cluster 3** |  |  |
| Alur | 10 | 6 | 1 | 17 |  |
| Lugbara | 10 | 3 | 0 | 13 |  |
| Banyuro | 1 | 1 | 1 | 3 |  |
| Kakwa | 1 | 2 |  | 3 |  |
| Kaliko |  | 1 |  | 1 |  |
| Kebu | 1 |  |  | 1 |  |
| Lendu | 2 |  |  | 2 |  |
| Ma'di |  | 1 |  | 1 |  |
| unknown | 2 | 1 |  | 3 |  |
| Other total (not Alur or Lugbara) | 7 | 6 | 1 | 14 |  |
| **Grand Total** | 27 | 15 | 2 | 44 |  |

## S2 Results. Do British and Ugandan mothers have different attitudes towards parenting and socialisation goals for their infants?

#### Importance mothers attributed to each socialisation goal

Table J shows the relative scores of importance that Ugandan and British mothers attributed to each socialisation goals item.

|  | |  | |  | |
| --- | --- | --- | --- | --- | --- |
| **Table J. Relative Scores of Importance for Each of the Socialisation Goals Items** | | | | | |
| **Item** | **Uganda (n = 41)** | | **UK**  **(n = 51)** | | **Total**  **(n = 92)** |
| Learn to control emotions | 0.15 | | -0.05 | | 0.04 |
| Learn to care for the well-being of others | 0.17 | | 0.12 | | 0.14 |
| Learn to obey adults | 0.92 | | -0.10 | | 0.36 |
| Develop independence | -0.38 | | 0.35 | | 0.02 |
| Develop self-confidence | -0.45 | | 0.52 | | 0.09 |
| Develop competitiveness | -0.41 | | -0.84 | | -0.65 |

This suggests that the relational socialisation goal item “learn to obey adults” discriminates well between the two samples and in the expected direction. The item “learn to control emotions” also appears to discriminate to some extent between Ugandan and British mothers. The relational socialisation goal “learn to care for the well-being of others” on the other hand seems to be valued to similar degrees by mothers from both cultural backgrounds.

With regards to the autonomous socialisation goals, the items “develop independence” and “develop self-confidence” both seem to be good discriminators between the two samples in the expected direction. The item “develop competitiveness” however, seems to have not been valued very much by mothers from both cultural backgrounds and therefore to not discriminate between the two samples.

## S3 Results. Does cultural group and infant age influence early life environment?

Tables K-R show full GLMM statistics for models investigating age and cultural group effects for specific variables of interest.

### Physical milestones

**Table K. Model parameters for physical milestone GLMMs.** The reference level for Group was Uganda. The reference level for physical milestones was ‘not able to’ (e.g. for Sit, the reference level was ‘not able to sit’). LRT = Likelihood Ratio Test. * indicates significance at *p*<.05 level, ** indicates significance at *p*<.01 level, *** indicates significance at *p*<.001 level.

|  | **Model parameters** | | | | | |
| --- | --- | --- | --- | --- | --- | --- |
| **Model (LRT Chi-Square)** | **Factor** | **Estimate** | **SE** | **Z** | **95% confidence interval** | ***p*** |
| Sit GLMM (*χ*^2^_(3)_=6.95, *p*=.008**) | (Intercept) | .870 | .461 | 1.89 | -.052 to 2.08 | .059 |
|  | Group | -1.30 | .458 | -2.85 | -2.39 to -.379 | .004** |
| Crawl GLMM (*χ*^2^_(3)_=12.7, *p*<.001***) | (Intercept) | 1.49 | .412 | 3.63 | .754 to 2.36 | <.001*** |
|  | Group | -1.39 | .419 | -3.31 | -2.24 to -.608 | <.001*** |
| Walk GLMM (*χ*^2^_(3)_=6.03, *p*=.014*) | (Intercept) | .139 | .406 | .343 | -.738 to 1.09 | .731 |
|  | Group | -1.07 | .504 | -2.12 | -2.48 to -.209 | .035* |

### Infant social environment - Caregivers

Ugandan infants had a higher number of carers in a day than in the UK (Table L; Figure Fa). There was no effect of infant age on the number of carers an infant would have during a day and no significant interaction between group and age.In Uganda, compared to in the UK, infants were significantly more likely to have a non-mother adult caregiver during their full-day follow (Table L; Figure Fb). There was no effect of age on how likely infants were to have a non-mother adult caregiver and no significant interaction between group and age. In Uganda, infant age had a significant effect on the likelihood that an infant was cared for by a child, and as Ugandan infants aged, they were more likely to have a carer who was a child (Table L; Figure Fc).

**Table L. Model parameters for Infant Caregivers GLMMs.** The reference level for Group was Uganda. The reference level for Adult Caregiver and Child Caregiver was ‘caregiver not a non-mother adult’ and ‘caregiver was not a child’ respectively. LRT = Likelihood Ratio Test. * indicates significance at *p* <.05 level, ** indicates significance at *p* <.01 level, *** indicates significance at *p* <.001 level.

|  | **Model parameters** | | | | | |
| --- | --- | --- | --- | --- | --- | --- |
| **Model (LRT Chi-Square)** | **Factor** | **Estimate** | **SE** | **Z** | **95% confidence interval** | ***p*** |
| Number of Caregivers GLMM (*χ*^2^_(2)_=106, *p*<.001***) | (Intercept) | .418 | .160 | 2.61 | .108 to .728 | .009** |
|  | Group | -1.59 | .347 | -4.57 | -2.33 to -.921 | <.001*** |
|  | Infant age | .010 | .014 | .694 | -.019 to .034 | .488 |
|  | Group *Infant Age | -.043 | .035 | -1.22 | -.115 to .029 | .223 |
| Non-mother Adult Caregiver GLMM (*χ*^2^ _(2)_=12.6, *p*=.005**) | (Intercept) | -3.38 | .278 | -12.2 | -3.94 to -2.79 | <.001*** |
|  | Group | -1.05 | .428 | -2.46 | -2.04 to -.231 | .014* |
|  | Infant age | .019 | .014 | 1.32 | -.010 to .044 | .186 |
|  | Group *Infant Age | .002 | .026 | .061 | -.052 to .059 | .952 |
| Child Caregiver UG GLMM (*χ*^2^ _(2)_=7.03, *p*=.008**) | (Intercept) | -2.28 | .175 | -13.1 | -2.65 to -1.93 | <.001*** |
|  | Infant age | .027 | .010 | 2.66 | .006 to .047 | .008** |

| 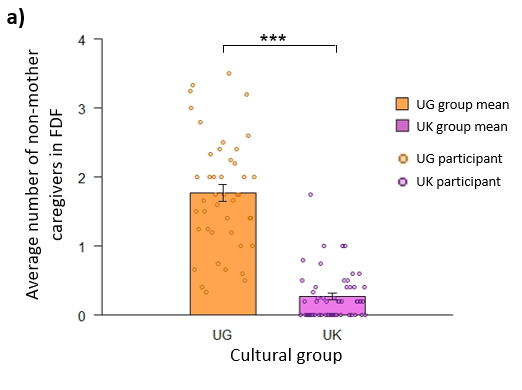 | 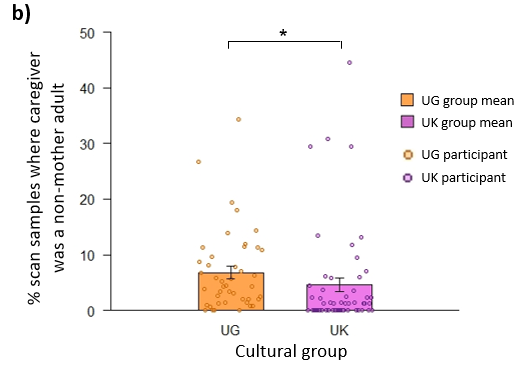 |
| --- | --- |
| 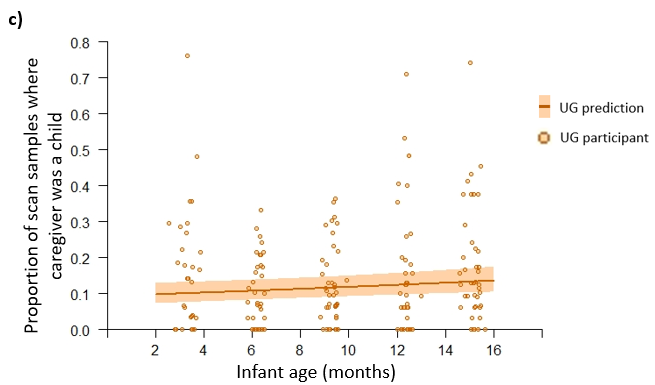 | |
| **Figure F. Graphs indicating significant effects found in caregiver GLMMS: a) Individual (dots) and group (bar) means of number of non-mother caregivers in a FDF with standard error bars; b) Individual (dots) and group means (bar) of proportion of scan samples where caregiver was a non-mother adult with standard error bars; c) Actual proportion of scan samples where individual Ugandan participants had a child caregiver (dots) and the expected probabilities of infants having a child caregiver given GLMM results (lines) as they age** **Shading around the line in c shows 95% confidence intervals.** UG=Ugandan, UK= British**.** * indicates significant group difference at <.05 level, *** indicates significant group difference at p<.001 level. | |

### Infant social environment - People in proximity of infant

As infants aged the likelihood of having a non-mother adult within five meters reduced (Table M; Figure Ga). There was no effect of group on the likelihood for there to be a non-mother adult in five meters of the infant and there was no significant interaction between group and age.

There was an interaction between group and infant age with how likely it was for there to be a child within five meters of the infant (Table M; Figure Gb). Whilst Ugandan infants were more likely to have a child in proximity than British infants across all ages, the likelihood of being in proximity to another child varied more with age in British infants than Ugandan infants, where proximity to another child was relatively stable as the infant aged. However, nine Ugandan participants and nine British participants were deemed overly influential in the GLMM indicating instability in the model. To further understand the effects of these participants, the GLMM was rerun without these participants. The model parameters were similar for the main effect of culture, however the interaction effect of age and culture was no longer significant (model including all participants: interaction *Est*=.036, *SE*=.013, *z*=2.88, *p*=.004; model without overly influential participants: interaction *Est*=.015, *SE*=.014, z=1.07, *p*=.284): there was no longer an age effect on the chances of having child in proximity in the UK when these participants were excluded (Figure Gb).

**Table M. Model parameters for individuals in proximity of infant GLMMs.** The reference level for Group was Uganda. The reference level for individuals in proximity was ‘not in 5 metres’. LRT = Likelihood Ratio Test.*** indicates significance at *p* <.001 level. ~** indicates an unstable effect with significance at *p* <.01 level (i.e. when model was run without overly influential participants this effect was no longer significant)

|  | **Model parameters** | | | | | |
| --- | --- | --- | --- | --- | --- | --- |
| **Model (LRT Chi-Square)** | **Factor** | **Estimate** | **SE** | **Z** | **95% confidence interval** | ***p*** |
| Number of people in 5 metre Proximity GLMM (*χ*^2^ _(2)_=3.99, *p=*.262) | (Intercept) | .299 | .091 | 3.28 | .119 to .478 | <.001*** |
|  | Group | -.047 | .126 | -.376 | -.296 to .201 | .707 |
|  | Infant age | -.008 | .005 | -1.65 | -.018 to .002 | .099 |
|  | Group *Infant Age | .013 | .008 | 1.73 | -.002 to .028 | .084 |
| Adults in 5 metre Proximity GLMM (*χ*^2^ _(2)_=22.2, *p<.001****) | (Intercept) | -.757 | .138 | -5.47 | -1.01 to -.489 | <.001*** |
|  | Group | .243 | .191 | 1.27 | -.123 to .612 | .203 |
|  | Infant age | -.033 | .008 | -4.11 | -.048 to -.018 | <.001*** |
|  | Group *Infant Age | .000 | .012 | .036 | -.022 to .023 | .972 |
| Children in 5 metre Proximity GLMM (all participants) (*χ*^2^ _(2)_=20.8, *p<.001****) | (Intercept) | -.052 | .213 | -.244 | -.459 to .355 | .807 |
|  | Group | -1.21 | .299 | -4.03 | -1.78 to -.623 | <.001*** |
|  | Infant age | -.002 | .007 | -.234 | -.016 to .014 | .815 |
|  | Group *Infant Age | .036 | .013 | 2.88 | .012 to .060 | .004~** |

| 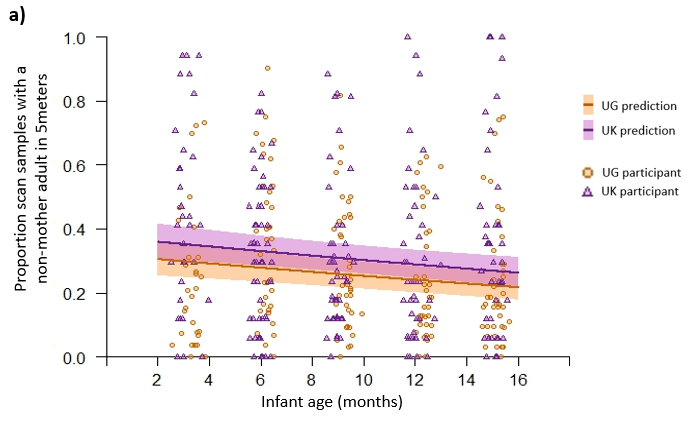 |
| --- |
| 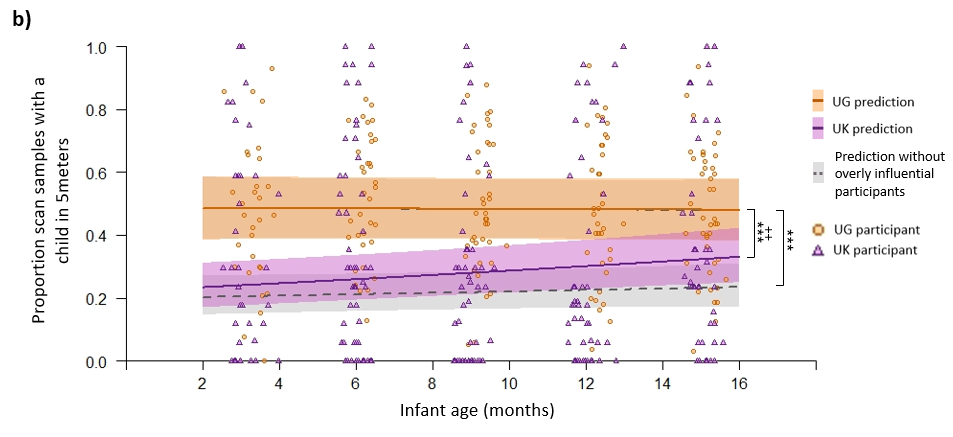 |
| **Figure G. Graphs indicating significant effects found in people in proximity GLMMS. a) Actual proportion of scan samples where there was a non-mother adult in 5m of the infant (circles/triangles) and the expected probabilities of this given GLMM results (lines) as infants age. b) Actual proportion of scan samples where there was a child in 5m of the infant (circles/triangles) and the expected probabilities of this given GLMM results (lines) as infants age.** UK=British, UG=Ugandan**.** Shading around the lines show 95% confidence intervals. For children in 5meters predications from full model (orange and purple shading and solid lines) is laid over the prediction for the model excluding overly influential participants (grey shading and dotted line). Prediction with and without influential participants for Ugandan sample is almost identical. Age effects in British participants disappear when overly influential participants are excluded. In both models there is a significant main effect of group. *** indicates significant main effect of group at p<.001 level. ++ indicates significant interaction at p<.01 level. |

### Infant social experience - Infant social activities

There was no group difference between Ugandan and British infants regarding how likely they were to be engaged in social activities (including play) and there was no significant interaction between group and infant age. However, as infants age, the chances that they would be engaged in a social activity reduced (Table N; Figure H).

**Table N. Model parameters for infant social activities GLMMs.** The reference level for Group was Uganda. The reference level for Social Play was ‘not engaged in play’. The reference level for Mother-Infant Contact during play was ‘not in contact’. The reference level for mother activities for infant was ‘not for infant’. The reference level for Social Activity was ‘not engaged in a social activity’. LRT = Likelihood Ratio Test. * indicates significance at *p* <.05 level, *** indicates significance at *p* <.001 level.

|  | **Model parameters** | | | | | |
| --- | --- | --- | --- | --- | --- | --- |
| **Model (LRT Chi-Square)** | **Factor** | **Estimate** | **SE** | **Z** | **95% confidence interval** | ***p*** |
| Social Play GLMM (*χ*^2^ _(2)_=69.2, *p<*.001***) | (Intercept) | -2.59 | .149 | -17.4 | -2.88 to -2.32 | <.001*** |
|  | Group | .464 | .192 | 2.41 | .082 to .845 | .016* |
|  | Infant age | -.011 | .013 | -.826 | -.038 to .014 | .409 |
|  | Group *Infant Age | .041 | .018 | 2.35 | .008 to .077 | .019* |
| Social Activities GLMM (*χ*^2^ _(2)_=39.7, *p<*.001***) | (Intercept) | -.947 | .090 | -10.5 | -1.13 to -.773 | <.001*** |
|  | Group | .124 | .128 | .966 | -.131 to .374 | .334 |
|  | Infant age | -.034 | .008 | -4.20 | -.049 to -.018 | <.001*** |
|  | Group *Infant Age | .014 | .012 | 1.20 | -.008 to .037 | .231 |

| 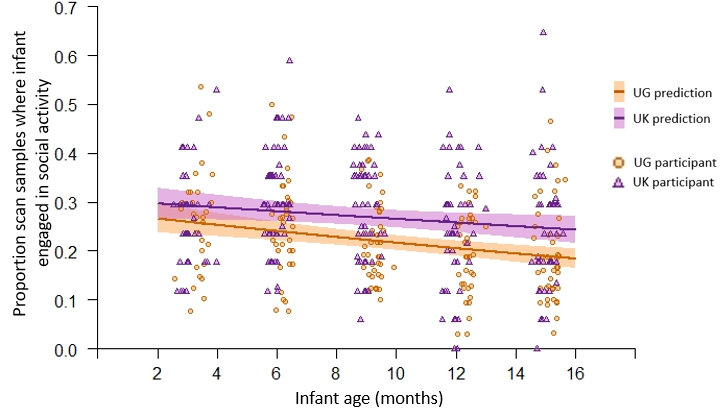  **Figure H. Graph showing actual proportion of scan samples where infant engaged in a social activity (circles/triangles) and the expected probabilities of this given GLMM results (lines) as they age.** UK=British, UG=Ugandan. Shading around the lines show 95% confidence intervals. The number of scan samples in the UK where infant activity was known was more standard across participants, hence why clusters are more commonly seen in the observed values for individual British participants. |
| --- |

### Infant social experience - Infant interaction partners

**Table O. Model parameters for infant social partners GLMMs.** The reference level for Group was Uganda. The reference level for non-mother adult and child social partners was ‘no’. LRT = Likelihood Ratio Test. ** indicates significance at *p*<.01 level, *** indicates significance at <.001 level. ~** indicates an unstable effect with significance at *p*<.01 level (i.e. when model was run without overly influential participants this effect was no longer significant).

|  | **Model parameters** | | | | | |
| --- | --- | --- | --- | --- | --- | --- |
| **Model (LRT Chi-Square)** | **Factor** | **Estimate** | **SE** | **Z** | **95% confidence interval** | ***p*** |
| Number of Social Partners GLMM (*χ*^2^ _(2)_=31.3, *p<*.001***) | (Intercept) | -3.29 | .197 | -16.7 | -3.68 to -2.96 | <.001*** |
|  | Group | -.260 | .274 | -.948 | -.794 to .251 | .343 |
|  | Infant age | -.001 | .018 | -.079 | -.034 to .033 | .937 |
|  | Group *Infant Age | .075 | .024 | 3.06 | .028 to .124 | .002** |
| Adult Social Partners GLMM (*χ*^2^ _(2)_=35.5, *p<*.001***) | (Intercept) | -2.69 | .374 | -7.20 | -3.41 to -2.04 | <.001*** |
|  | Group | .175 | .455 | .384 | -.625 to 1.11 | .701 |
|  | Infant age | -.065 | .035 | -1.84 | -.139 to .001 | .065 |
|  | Group *Infant Age | .129 | .042 | 3.06 | .052 to .211 | .002~** |
| Child Social Partners GLMM (*χ*^2^ _(2)_=61.8, *p<*.001***) | (Intercept) | -2.52 | .268 | -9.40 | -3.12 to -2.01 | <.001*** |
|  | Group | -1.36 | .411 | -3.31 | -2.21 to -.557 | <.001*** |
|  | Infant age | .062 | .022 | 2.86 | .019 to .109 | .004** |
|  | Group *Infant Age | .119 | .034 | 3.51 | .055 to .188 | <.001*** |

**Proximity between infant and caregivers – Mother proximity during the day**

**Table P. Model parameters for mother-infant proximity, contact, and contact during play GLMMs.** The reference level for Group was Uganda. The reference level for mother-infant contact was ‘not in contact’. LRT = Likelihood Ratio Test. * indicates significance at *p*<.05 level, *** indicates significance at *p*<.001 level.

|  | **Model parameters** | | | | | | |
| --- | --- | --- | --- | --- | --- | --- | --- |
| **Model (LRT Chi-Square)** | **Factor** | **Estimate** | **SE** | **Z** | **95% confidence interval** | ***p*** |  |
| Proximity with mother: all occasions GLMM (*χ*^2^ _(2)_=117, *p<*.001***) | (Intercept) | .976 | .130 | 7.49 | .723 to 1.22 | <.001*** |  |
|  | Group | .926 | .192 | 4.82 | .561 to 1.31 | <.001*** |  |
|  | Infant age | -.054 | .007 | -7.31 | -.069 to -.039 | <.001*** |  |
|  | Group*Infant Age | .004 | .013 | 0.297 | -.020 to .028 | .767 |  |
| Contact with mother: all occasions GLMM (*χ*^2^ _(2)_=291, *p<*.001***) | (Intercept) | .007 | .114 | .060 | -.221 to .233 | .952 |  |
|  | Group | -.094 | .162 | -.577 | -.409 to .220 | .564 |  |
|  | Infant age | -.088 | .008 | -11.7 | -.104 to -.073 | <.001*** |  |
|  | Group *Infant Age | -.027 | .012 | -2.19 | -.051 to -.003 | .029* |  |
| Contact with mother: during play GLMM (*χ*^2^ _(2)_=55.9, *p<*.001***) | (Intercept) | 2.75 | .668 | 4.11 | 1.57 to 4.18 | <.001*** |  |
|  | Group | -1.75 | .733 | -2.39 | -3.24 to -.492 | .017* |  |
|  | Infant age | -.116 | .059 | -1.98 | -.232 to -.014 | .047* |  |
|  | Group *Infant Age | -.008 | .066 | -.128 | -.126 to .123 | .898 |  |

###

### Additional models to test mother proximity to the infant during the day

When considering all occasions, mothers and infants in the UK were more likely to be in five meters of one another than dyads in Uganda. In order to understand whether this was a feature of caregiving being more shared in Uganda than the UK, or whether it is a feature of mother’s caregiving style, we ran the same GLMM reduced to only scan samples where the mother was the caregiver and the same group and age effects were found (Table Q; Figure I).

**Table Q.** Model parameters for mother-infant proximity and body contact when the mother was the carer GLMMs. The reference level for Group was UK. The reference level for mother-infant contact was ‘not in contact’. LRT = Likelihood Ratio Test. * indicates significance at <.05 level, *** indicates significance at <.001 level.

|  | **Model Parameters** | | | | | |
| --- | --- | --- | --- | --- | --- | --- |
| **Model** (LRT Chi-Square) | **Factor** | **Estimate** | **SE** | ***Z*** | **95% confidence intervals** | ***P*** |
| Proximity with mother: when mother carer GLMM (*χ*^2^ _(2)_=51.3, *p<*.001***) | (Intercept) | 1.54 | .147 | 10.5 | 1.25 to 1.83 | <.001*** |
|  | Group | .546 | .209 | 2.61 | .136 to .961 | .009** |
|  | Infant age | -.042 | .009 | -4.47 | -.061 to -.024 | <.001*** |
|  | Group*Infant Age | -.008 | .014 | -.583 | -.037 to .020 | .560 |
| Contact with mother: when mother carer GLMM (*χ*^2^ _(2)_=315, *p<*.001***) | (Intercept) | .556 | .118 | 4.71 | .324 to .789 | <.001*** |
|  | Group | -.557 | .166 | -3.36 | -.883 to -.232 | <.001*** |
|  | Infant age | -.097 | .008 | -11.6 | -.114 to -.081 | <.001*** |
|  | Group*Infant Age | -.021 | .013 | -1.6 | -.046 to .004 | .103 |

| 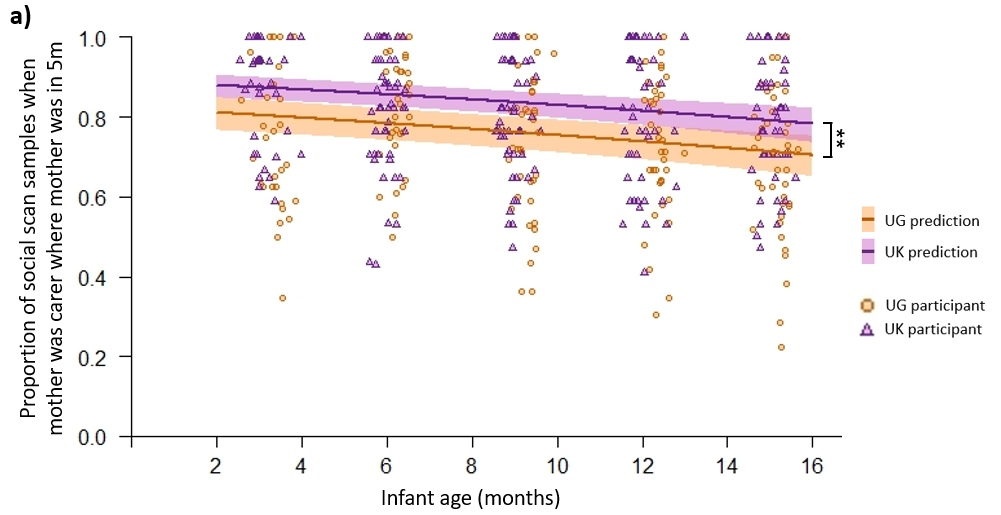 |
| --- |
| 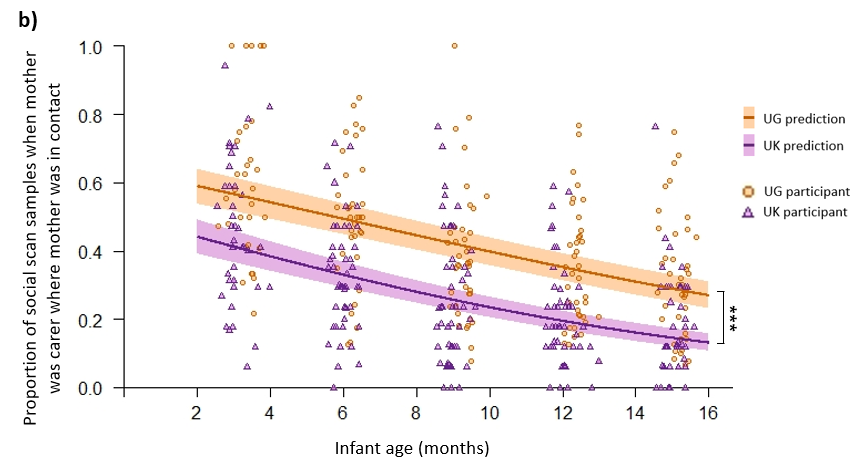 |
| **Figure I. Graphs indicating significant effects found in mother-infant distance when mother was caregiver GLMMS. a) Actual proportion of scan samples where when mother was carer that the mother was in 5m of the infant (circles/triangles) and the expected probabilities of this given GLMM results (lines) as infants age. b) Actual proportion of scan samples where when mother was carer that the mother was in contact with the infant (circles/triangles) and the expected probabilities of this given GLMM results (lines) as infants age.** UK=British, UG=Ugandan. Shading around the lines show 95% confidence intervals. ** indicates significant main effect of group at p<.01 level. *** indicates significant main effect of group at p<.001 level. |

### Proximity at night: Infant sleeping arrangements

In the UK, infants were less likely to share a bedroom at night as they aged (Table R; Figure J)

| 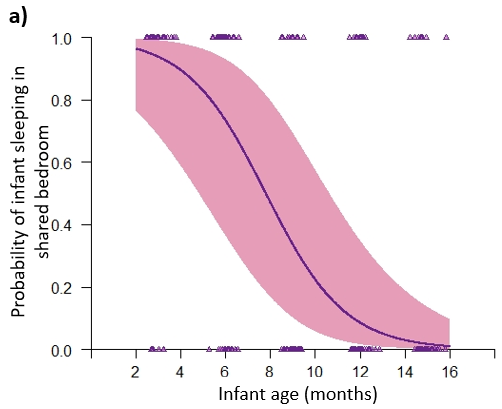 |
| --- |
| **Figure J. Graph showing predicted probability of British infants sharing a bedroom across age. Triangles indicate whether individuals did (1) or didn’t (0) share a room. Shaded area indicates 95% confidence interval.** |

**Table R. Model parameters for UK infant sleeping arrangement GLMMs.** The reference level for shared bedroom was ‘slept in own room’. LRT = Likelihood Ratio Test. *** indicates significance at *p*<.001 level.

|  | **Model parameters** | | | | | |
| --- | --- | --- | --- | --- | --- | --- |
| **Model (LRT Chi-Square)** | **Factor** | **Estimate** | **SE** | **Z** | **95% confidence interval** | ***p*** |
| Shared Bedroom GLMM (*χ*^2^ _(2)_=59.4, *p=<.001****) | (Intercept) | 4.41 | 1.25 | 3.52 | 2.28 to 7.44 | <.001*** |
|  | Infant age | -.565 | .123 | -4.60 | -.869 to -.364 | <.001*** |

## S4 Results. Influential participants

In order to test model stability, overly influential participants were identified (see Table S) and models were then run without participants which were deemed to be overly influential. Cases of influential participants did not appear to be affected by whether the infant was a twin, or whether their mother was an immigrant. The median for the number of models where a twin was identified as influential, and number of models where non-twins were identified as influential was 2 in both cases. Likewise, the median for the number of models where infants of non-immigrants were deemed influential, and number of models where infants of immigrants was 2 in both cases.

Table T shows the original model parameters and the model parameters when excluding these overly influential participants for the five models where interpretation was impacted by removal of influential participants.

**Table S. The number of over-influential participants identified in each model.**

| **Model** | **Number overly influential participants** | |
| --- | --- | --- |
|  | Ugandan | British |
| Sit GLMM | 1 | 2 |
| Crawl GLMM | 3 | 2 |
| Walk GLMM | 3 | 3 |
| Environment Exploration GLMM | 2 | 2 |
| Number of Carers GLMM | 1 | 3 |
| Adult Carer GLMM | 10 | 17 |
| Child Carer GLMM | 9 | n/a |
| Proximity with Mother GLMM | 7 | 6 |
| Number of people in Proximity GLMM | 5 | 9 |
| Adults in Proximity GLMM | 9 | 11 |
| Children in Proximity GLMM | 9 | 9 |
| Shared Bedroom GLMM | n/a | 1 |
| Shared Bed GLMM | n/a | 5 |
| Contact with Mother GLMM | 8 | 3 |
| Mother Activities for Infant GLMM | 1 | 2 |
| Social Play GLMM | 2 | 1 |
| Contact with mother during play GLMM | 2 | 4 |
| Social Activities GLMM | 2 | 2 |
| Number of Social Partners GLMM | 2 | 4 |
| Adult Social Partners GLMM | 3 | 3 |
| Child Social Partners GLMM | 2 | 1 |

**Table T.** Model parameters from the original GLMMs and the models rerun excluding these overly influential participants for models where interpretation was impacted by removal of influential participants.

|  |  | ***Parameters for full model*** | | | | ***Parameters for model excluding overly influential participants*** | | | |
| --- | --- | --- | --- | --- | --- | --- | --- | --- | --- |
| **Model** | **Factor** | **Estimate** | **SE** | ***Z*** | ***P*** | **Estimate** | **SE** | ***Z*** | ***P*** |
| Number of people in Proximity GLMM | (Intercept) | .299 | .091 | 3.28 | <.001*** | .315 | .094 | 3.37 | <.001*** |
|  | Group | -.047 | .126 | -.376 | .707 | .009 | .130 | .070 | .944 |
|  | Infant age | -.008 | .005 | -1.65 | .099 | -.013 | .005 | -2.38 | .018* |
|  | Group *Infant Age | .013 | .008 | 1.73 | .084 | .007 | .008 | .875 | .382 |
| Children in Proximity GLMM | (Intercept) | -.052 | .213 | -.244 | .807 | -.051 | .209122 | -.243 | .808 |
|  | Group | -1.21 | .299 | -4.03 | <.001*** | -1.35 | .295 | -4.57 | <.001*** |
|  | Infant age | -.002 | .007 | -.234 | .815 | -.002 | .008 | -.240 | .81 |
|  | Group *Infant Age | .036 | .013 | 2.88 | .004** | .015 | .014 | 1.07 | .284 |
| Shared Bed GLMM | (Intercept) | -8.54 | 1.86 | -4.58 | <.001*** | -14.6 | 5.07 | -2.89 | .004** |
|  | Infant age | -.368 | .154 | -2.39 | .017* | -.014 | .404 | -.035 | .972 |
| Contact with Mother GLMM | (Intercept) | .007 | .114 | .060 | .952 | -.014 | .124 | -.114 | .909 |
|  | Group | -.094 | .162 | -.577 | .564 | .032 | .171 | .186 | .853 |
|  | Infant age | -.088 | .008 | -11.7 | <.001*** | -.091 | .008 | -10.8 | <.001*** |
|  | Group *Infant Age | -.027 | .012 | -2.19 | .029* | -.038 | .013 | -2.87 | .004 |
| Adult Social Partners GLMM | (Intercept) | -2.69 | .374 | -7.20 | <.001*** | -3.15 | .419 | -7.51 | <.001*** |
|  | Group | .175 | .455 | .384 | .701 | .628 | .496 | 1.27 | .205 |
|  | Infant age | -.065 | .035 | -1.84 | .065 | -.031 | .039 | -.797 | .425 |
|  | Group *Infant Age | .129 | .042 | 3.06 | .002** | .088 | .046 | 1.90 | .058 |

## Supplementary references

1. R_Core_Team. R: A Language and Environment for Statistical Computing [Internet]. Vienna, Austria: R Foundation for Statistical Computing; 2020. Available from: https://www.r-project.org/

2. Schafer J, Opgen-Rhein R, Zuber V, Ahdesmaki M, Silva APD, Strimme K. corpcor: Efficient Estimation of Covariance and (Partial) Correlation [Internet]. 2017. Available from: https://cran.r-project.org/package=corpcor

3. Bernaards CA, Jennrich RI. Gradient projection algorithms and software for arbitrary rotation criteria in Ffactor analysis. Educ Psychol Meas. 2005;(65):676–96.

4. Revelle W. psych: Procedures for Psychological, Psychometric, and Personality Research [Internet]. Evanston, Illinois: Northwestern University; 2020. Available from: https://cran.r-project.org/package=psych

5. Kaiser HF. The application of electronic computers to factor analysis. Educ an Psychol Meas. 1960;20(1):141–51.

6. Field A, Miles J, Field Z. Discovering statistics using R. SAGE Publications; 2012.
